# DNA co-methylation modules in postmortem prefrontal cortex tissues of European Australians with alcohol use disorders

**DOI:** 10.1038/srep19430

**Published:** 2016-01-14

**Authors:** Fan Wang, Hongqin Xu, Hongyu Zhao, Joel Gelernter, Huiping Zhang

**Affiliations:** 1 Department of Psychiatry, Yale University School of Medicine, New Haven, CT, USA; 2 Department of Genetics, Yale University School of Medicine, New Haven, CT, USA; 3 Department of Neurobiology, Yale University School of Medicine, New Haven, CT, USA; 4 VA Medical Center, VA Connecticut Healthcare System, West Haven, CT, USA; 5 Department of Biostatistics, Yale University School of Public Health, New Haven, CT, USA

## Abstract

DNA methylome alterations in the prefrontal cortex (PFC) may contribute to risk for alcohol use disorders (AUDs). We examined postmortem PFC DNA methylomes of 16 male and seven female pairs of AUD and control subjects using Illumina’s HumanMethylation450 BeadChip assays. In male AUD subjects, 1,812 CpGs (1,099 genes) were differentially methylated (9.5 × 10^−9^ ≤ *P*_nominal_ ≤ 7.2 × 10^−4^, q < 0.05). In females, no CpGs were associated with AUDs after multiple testing correction (q > 0.05). Twenty-one AUD-associated co-methylation modules were identified in males by co-methylation analysis. The 1,812 CpGs were over-presented by two AUD-associated co-methylation modules (M_turquoise_: 1,048 CpGs/683 genes; M_blue_: 429 CpGs/304 genes) (*P*_hyper_ ≤ 0.001). Biological processes enriched for genes in these two modules included neural development and transcriptional regulation. Genes mapped by CpGs in these two modules were enriched in genome-wide association study-identified genes with variants associated with four substance dependence phenotypes or five psychiatric disorders. Additionally, 106 of the 1,812 CpGs were mapped to 93 genes (e.g., AUD-associated genes *GRIK3, GRIN2C*, and *GABRA1*) with differential expression in postmortem PFC of male AUD subjects. Our study demonstrates that DNA methylation alterations in the PFC are associated with (and might result in) increased risk of AUDs, and there was a complex DNA methylation-gene expression relationship.

Alcohol use disorders (AUDs), including alcohol abuse or dependence, are prevalent and associated with a variety of medical and social problems. Despite the high prevalence of AUDs (over 8%)^1^, their molecular mechanisms are not well understood. Given their heterogeneous nature, AUDs are likely to be caused by variation in multiple genes and by gene-gene and gene-environment interactions^2^. Environmental factors alone (e.g., chronic alcohol consumption) may also lead to alcohol tolerance or dependence through neuroadaptations. Increasing evidence suggests that drugs of abuse and drug-associated cues can alter gene expression in neurons, and this process may be mediated by epigenetic mechanisms (e.g., DNA methylation)^3^. The development of AUDs may result from neuroadaptations involving epigenetic modifications after long-term alcohol consumption^4^.

Several candidate gene studies^5^,^6^,^7^,^8^,^9^, including ours^10^,^11^, have shown altered DNA methylation in peripheral blood of AUD subjects. The only published genome-wide DNA methylation study of AUDs showed widespread DNA methylation changes in lymphoblastoid cells derived from peripheral blood lymphocytes^12^. The extent to which DNA methylation in peripheral blood reflects that in the brain is not well established. Our recent study^13^, using the drinking-in-the-dark (DID) mouse model, demonstrated that alcohol exposure resulted in altered DNA methylation in the promoter region of the serotonin receptor 3a gene (*Htr3a*) in the peripheral blood, while loci in brain reward circuits showed a wide range of alterations in the methylation of this gene.

To date, only two published studies have analyzed AUD-associated DNA methylation changes in postmortem brains. One study examined methylation levels of CpGs in the prodynorphin gene (*PDYN*) and reported altered methylation of three SNP-CpGs in postmortem prefrontal cortex (PFC) of AUD subjects^14^. Another study examined global methylation alterations in postmortem brains of AUD subjects using methylated DNA immunoprecipitation and microarray^15^, but the sample size was small (10 AUD cases and 10 controls) and the differential methylation at individual CpGs was not directly measured.

The above DNA methylation studies were limited as follows. First, most of the studies examined DNA methylation changes only in peripheral blood cells of AUD subjects; but DNA methylation patterns in peripheral blood may not reflect those in brain reward regions. Second, most published studies analyzed AUD-associated DNA methylation changes only in the promoter region of candidate genes; while intragenic (gene body or 3′ UTR)^16^,^17^ and intergenic^18^,^19^ DNA methylation can also impact gene transcription. Third, although two studies examined DNA methylomic changes in AUD subjects, DNA samples for one study^12^ were extracted from lymphoblastoid cells and the sample size for another study^15^ was small. To understand the molecular basis of chronic alcohol consumption-induced neuroadaptations, a systematic analysis of methylomic alterations in reward-related brain regions (e.g., the PFC) of AUD subjects is required.

The PFC functions in planning cognitive performance and moderating social behaviors and has been linked to rewarding pathways and alcohol-seeking behaviors^20^,^21^. Thus, in the present study, we examined genome-wide DNA methylation alterations in postmortem PFC of 16 pairs of male and seven pairs of female AUD and control subjects (46 European Australians in total). We identified differentially methylated CpGs (or CpG clusters) in AUD subjects, discovered biological pathways in which genes harboring these CpGs are involved, analyzed the enrichment of differentially methylated genes in substance dependence and psychiatric disorders-associated genes, and examined the correlation of differentially methylated CpGs and differentially expressed genes in the PFC.

## Results

### Differentially methylated CpGs in AUD subjects

**Figure 1:**
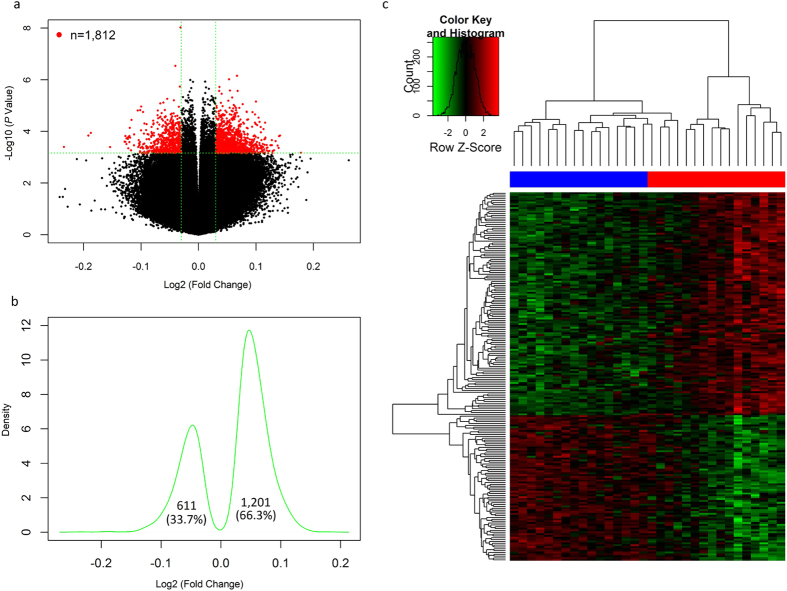
Differentially methylated CpGs in male subjects with alcohol use disorders (AUDs). (**a**) Volcano plot of effect size [log_2_(fold changes)] against −log_10_(*P* values) of 434, 015 CpGs. The red dots represent 1,812 CpGs with q ≤ 0.05 (the horizontal green dash line) and the absolute value of log_2_(fold change) >0.03 (two vertical green dash lines), and the black dots represent non-significant CpGs. (**b**) Kernel density plotting of log_2_(fold changes) of 1,812 significant CpGs. The asymmetric plot indicates that a greater proportion (66.3%) of CpGs were hypermethylated in AUD subjects. (**c**). Hierarchical clustering of the 32 male subjects using a heatmap based on methylation levels of the above 1,812 CpGs (adjusted for age and PMI). The 32 male subjects were clustered into two distinct groups that were consistent with their actual AUD status (the red color: 16 male AUD patients; the blue color: 16 male healthy control subjects). The colors in the heatmap indicate CpG methylation levels (green to red: low to high methylation levels).

In male AUD subjects, 87,588 (20.4%) of the 430,407 CpGs (that remained after preprocessing Illumina 450 K methylation array data) showed differential methylation (*P*_nominal_ = 9.5 × 10^−9^ − 0.05), and the associations of 1,812 CpGs and AUDs survived correction for multiple testing (9.5 × 10^−9^ ≤ *P*_nominal_ ≤ 7.2 × 10^−4^ and q < 0.05) (Fig. 1a). Kernel density plotting of log_2_ (fold changes) of these 1,812 CpGs showed that a greater proportion (1,201/1,812 = 66.3%) of them were hypermethylated in male AUD subjects (Fig. 1b). Hierarchical clustering analysis, based on normalized methylation levels of these 1,812 CpGs and adjusted for age and postmortem interval (PMI), indicated that 32 male subjects (16 male AUD and 16 control subjects) included in this study were clustered into two distinct subgroups that were consistent with their AUD status (Fig. 1c). Of the 1,812 CpGs, 1,350 CpGs were located in or near genes (611 in promoters, 644 in gene bodies, and 95 in 3′UTRs) and 462 were located in intergenic regions. The top 20 hits are listed in Table 1. The three most significant CpGs were cg19099433 in the gene body of the allantoicase gene (*ALLC*) involved in the uric acid degradation pathway, cg16460727 in the promoter of the complexin 2 gene (*CPLX2*) involved in synaptic vesicle exocytosis, and cg11938646 in the promoter of the U42A small nucleolar RNA (snoRNA) gene (*SNORD42A*) involved in directing site-specific 2′-O-methylation of substrate RNAs. In the smaller sample of female AUD subjects, 22,091 (5.1%) of the above 430,407 CpGs showed differential methylation (*P*_nominal_ = 9.8 × 10^−7^ − 0.05). There were 154 CpGs with *P* < 1.0 × 10^−3^, and the three top hits were cg22707691 (*P*_nominal_ = 9.8 × 10^−7^) located in the 3′ UTR region of the NAD Kinase gene (*NADK*) which participates in the NAD^+^ kinase activity and two intergenic CpGs (cg19901523: *P*_nominal_ = 1.7 × 10^−5^; cg22788657: *P*_nominal_ = 2.4 × 10^−5^). However, none of them withstood correction for multiple testing (q > 0.05) (Supplemental Fig. S1).

### AUD-associated DNA methylation modules in male subjects

Since significant results were obtained from male subjects and more male subjects were included in this study (32 males *vs*. 14 females), we performed co-methylation analysis only in male subjects. AUD-associated CpGs identified in male subjects were first assigned into different modules (a module is defined as a group of co-methylated CpGs) by examining their pair-wise correlations. Then, the first principle component (PC1) of each module, which was calculated using normalized methylation levels of all CpGs within a module and represented the average methylation level of a module, was used to examine AUD-module associations. Under the default setting of the weighted gene co-expression network analysis (WGCNA) program^22^, the above 87,588 nominally significant CpGs (*P*_nominal_ < 0.05) identified in male AUD subjects were clustered into 21 modules (Supplemental Fig. S2). In each module (median size: 211 CpGs), CpGs were significantly correlated in their methylation levels. Those CpGs, which were not significantly correlated in their methylation levels, were assigned to the grey module (containing 9,615 CpGs). As expected, the overall methylation pattern (represented by the ME, i.e., the first principle component) of all 22 modules (including the grey module) was significantly correlated with AUDs. The top three modules (consisting of significantly correlated CpGs) were the green module (M_green_ with 1,890 CpGs: r = 0.75, *P*_*correlation*_ = 9.8 × 10^−7^), the light cyan module (M_lightcyan_ with 161 CpGs: r = 0.71, *P*_*corr*_ = 6.2 × 10^−6^), and the pink module (M_pink_ with 289 CpGs: r = 0.69, *P*_*corr*_ = 1.3 × 10^−5^).

**Figure 2:**
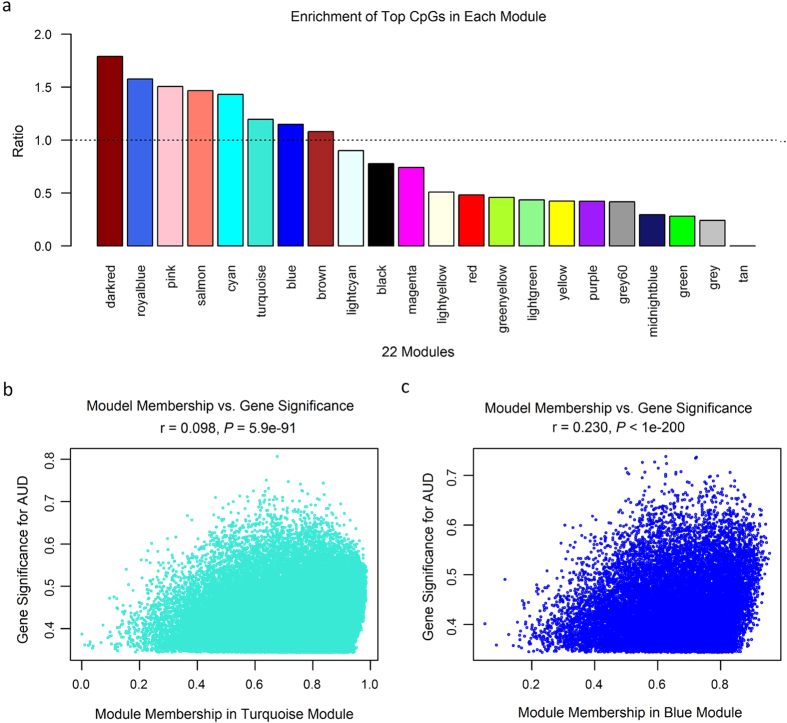
Alcohol use disorder (AUD)-associated CpG methylation modules. (**a**) Enrichment of the top 1,812 CpGs (identified in male AUD subjects) in each of the 22 modules (including the grey module). The ratio was calculated as the observed number of the top CpGs over the expected number of the top CpGs. (**b,c**). Correlation of the gene significance (GS) and the module membership (MM) in two large modules (the turquoise module and the blue module).

We further examined whether the top 1,812 CpGs identified in male AUD subjects were over-represented in any of the above 22 modules by hypergeometric-based tests. We calculated the ratio of the number of the observed top CpGs (n.top) over the expected number of CpGs in each module given the module size. Eight modules with a ratio of more than 1 were identified (M_darkred_: size = 81, n.top = 3, ratio = 1.8, *P*_hyper_ = 0.087; M_royalblue_: size = 92, n.top = 3, ratio = 1.6, *P*_hyper_ = 0.124; M_pink_: size = 289, n.top = 9, ratio = 1.5, *P*_hyper_ = 0.080; M_salmon_: size = 198, n.top = 6, ratio = 1.5, *P*_hyper_ = 0.119; M_cyan_: size = 169, n.top = 5, ratio = 1.4, *P*_hyper_ = 0.140; M_turquoise_: size = 42,389, n.top = 1,048, ratio = 1.4, *P*_hyper_ = 1.7 × 10^−16^; M_blue_: size = 18,061, n.top = 429, ratio = 1.1, *P*_hyper_ = 0.001; M_brown_: size = 8,469, n.top = 189, ratio = 1.1, *P*_hyper_ = 0.126) (Fig. 2a). The two larger modules (M_turquoise_ and M_blue_) showed significant enrichment (*P*_hyper_ ≤ 0.001) of the 1,812 top CpGs that were differentially methylated in male AUD subjects. For these two larger modules, we assessed the correlation of two parameters, i.e., the gene significance (GS) and the module membership (MM). As displayed in Fig. 2b,c, a significant positive correlation between the GS and the MM was observed in these two larger modules, suggesting that AUD-associated CpGs were also the most important (central) elements of the module for AUDs.

### Biological processes enriched for genes mapped by CpGs in modules M_turquoise_ and M_blue_

1,048 CpGs in the turquoise module (M_turquoise_) were mapped to 683 unique genes, and 429 CpGs in the blue module (M_blue_) were mapped to 304 unique genes. These two sets of genes were subjected to DAVID^23^ for functional annotation. As shown in Supplementary Table S1, highly enriched biological progresses observed in M_turquoise_ included: cell projection organization (fold = 2.5, *P* = 3.7 × 10^−6^), cell projection morphogenesis (fold = 2.6, *P* = 7.7 × 10^−5^), and neuron development (fold = 2.3, *P* = 9.8 × 10^−5^). Highly enriched biological progresses observed in M_blue_ included: rhythmic process (fold = 4.6, *P* = 7.1 × 10^−4^), positive regulation of transcription (fold = 2.4, *P* = 2.4 × 10^−3^), and DNA-dependent positive regulation of macromolecule metabolic process (fold = 1.9, *P* = 2.5 × 10^−6^).

### Genes mapped by CpGs in modules M_turquoise_ and M_blue_ were enriched in GWAS-identified genes

Gene set enrichment analysis (GSEA) was performed to examine the over-representation of differentially methylated genes by GWAS-identified genes mapped by SNPs associated with four types of substance dependence phenotypes [alcohol dependence (AD)^24^, cocaine dependence (CD)^25^, opioid dependence (OD)^26^, or nicotine dependence (ND)^27^] or five psychiatric disorders [attention deficit hyperactivity disorder (ADHD), autism spectrum disorders (ASD), bipolar disorder (BPD), major depressive disorder (MDD), or schizophrenia (SCZ)]^28^,^29^,^30^,^31^,^32^, given the pleiotropic effects of genetic variants (or epigenetic changes) on various neurodevelopmental, psychiatric, and neurological outcomes^32^,^33^,^34^. Table 2 summarizes the enrichment analysis results of genes mapped by CpGs in the above two large CpG modules (M_turquoise_: 1,048 CpGs mapped to 683 unique genes; M_blue_: 429 CpGs mapped 304 unique genes). Genes mapped by CpGs in both modules were highly enriched in GWAS-identified genes harboring SNPs that were associated with substance dependence or psychiatric disorders. When GWAS-identified genes were limited to those genes that were expressed in human PFC tissues^35^, similar results were observed (Table 2).

### Transcriptional effect of DNA methylation changes

**Figure 3:**
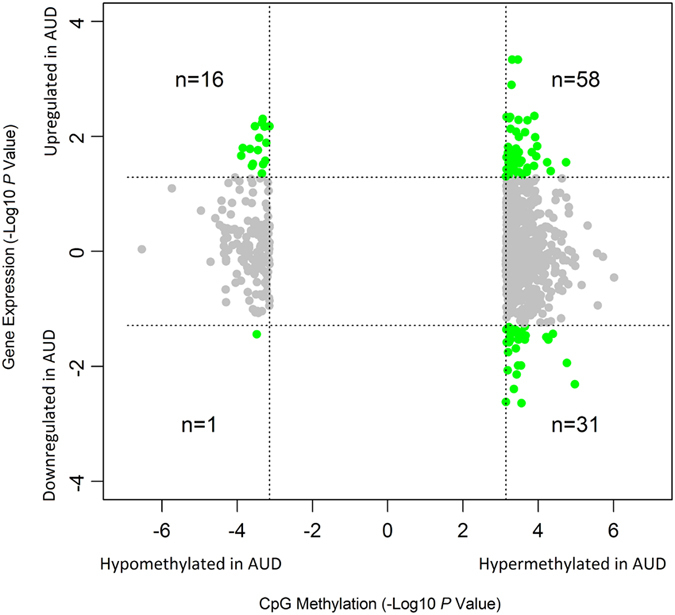
Pairs of hypo- or hypermethylated CpGs and up- or downregulated genes in postmortem PFC of male AUD subjects. Vertical and horizontal dash lines indicate significantly changed CpG methylation and gene expression, respectively.

The correlation of the above 22 AUD-associated CpG modules (including the grey module) and five AUD-associated gene expression modules^35^ (refer to “Materials and Methods”) was analyzed, and the results are presented in Supplementary Table S2. Two large AUD-associated CpG modules (M_turquoise_ and M_blue_) were significantly correlated with all five AUD-associated gene expression modules except the correlation of CpG module M_turquoise_ and gene expression module E_brown_. Of interest, those AUD-associated CpGs modules in which the top 1,812 CpGs were not enriched (refer to Fig. 2) were also correlated with some of the five AUD-associated gene expression modules (e.g., the M_red_-E_turquoise_ pair). Additionally, we analyzed the correlation of methylation levels of individual CpGs and expression levels of individual genes. Of the top 1,812 CpGs identified in male AUD subjects, 106 were mapped to 93 host genes that were differentially expressed in postmortem PFC of male AUD subjects (Fig. 3). These 106 CpG methylation-gene expression pairs were divided into four subgroups: (1) 58 pairs of hypermethylated CpGs and upregulated genes; (2) 31 pairs of hypermethylated CpGs and downregulated genes; (3) 16 pairs of hypomethylated CpGs and upregulated genes; and (4) one pair of a hypomethylated CpG and a downregulated gene (Table 3). The first two subgroups (hypermethylated CpGs and paired up- or down-regulated genes) were presented in Supplemental Table S3, and later two subgroups (hypomethylated CpGs and paired up- or down-regulated genes) were presented in Supplemental Table S4. Several genes previously identified to be AUD-related, such as *GRIK3, GRIN2C*, and *GABRA1*, were contained in the 106 CpG methylation-gene expression pairs. Additionally, genes involved in these 106 CpG methylation-gene expression pairs potentially participate in *Biological Processes*, such as cell morphogenesis, locomotion, differentiation, and homeostasis as well as neuron development (Supplemental Table S5).

## Discussion

In this study, we presented data demonstrating DNA methylome alterations in the PFC of AUD subjects (particularly in male AUD subjects). Most of the AUD-associated CpGs identified in male AUD subjects were significantly correlated, forming CpG clusters (or modules), in which the overall methylation pattern was also highly correlated with AUDs. Some of the genes mapped by CpGs in the module were involved in neural development and transcriptional regulation. Additionally, those genes mapped by CpGs contained in AUD-associated CpG modules were over-represented by GWAS-identified genes with variants associated with substance (alcohol, cocaine, opioid, or nicotine) dependence or psychiatric disorders (ADHD, ASP, BPD, MDD, or SCZ). The major findings of this study are discussed below.

First, we observed altered DNA methylation patterns in the PFC of AUD subjects (mainly male AUD subjects). To profile AUD-associated methylome alterations in reward-related brain regions, we used a set of postmortem PFC samples that were unique in two aspects: (1) AUD subjects did not have co-morbid drug dependence or major psychotic disorders, and thus DNA methylation changes in AUD subjects were more likely to have resulted from chronic alcohol consumption (or alternatively, be predisposing to this trait); and (2) AUD cases and healthy controls were well-matched to minimize the influence of confounding factors (such as sex, age, and PMI) on DNA methylation. When analyzing DNA methylation data from all 23 pairs of samples (i.e., 23 AUD cases *vs*. 23 matched healthy controls), no CpG sites showed genome-wide significant results (Supplementary Fig. S1a), although in the absence of confounding sex effects, our sample was expected to have sufficient power [the observed power (one-tail hypothesis) = 84.7%, supposing the observed effect size (Cohen’s d) is 0.8, the probability level is 0.05, and the sample size is 46] to identify differentially methylated CpGs. Given our recent findings concerning sex-biased effects on methylome patterns of CpGs located on either sex or autosomal chromosomes^36^, we proceeded to examine AUD-associated DNA methylation alterations in males and female separately. One thousand eight hundred and twelve CpGs (mapped to 1,099 genes) were associated with AUDs in male subjects (16 male AUD cases *vs*. 16 male controls) and the associations survived correction for multiple tests (Fig. 1), but no genome-wide significant results were obtained from the female subjects (7 female AUD cases *vs*. 7 female controls) (Supplementary Fig. S1b). The negative findings in female subjects is possibly due to (1) small sample size and consequent lack of statistical power [the observed power (one-tail hypothesis) = 40.1%, supposing the observed effect size (Cohen’s d) is 0.8, the probability level is 0.05, and the sample size is 14] to identify AUD-associated CpGs; and/or (2) the lower severity of alcohol consumption in AUD women compared to AUD men [this was reflected by less alcohol daily use in women (162.8 ± 79.5 g) compared to men (165.6 ± 84.2 g)], and thus DNA methylation alterations in AUD women may have been lower in magnitude, further compromising power. However, no significant overlap was observed between the top 1,000 CpGs identified in male and female AUD subjects (Enrichment fold = 0.87, *P*_hypergeometric_ = 0.329), suggesting that alcohol consumptions might induce different methylation patterns in males and females. It should be noted that individual alcohol consumption might confound DNA methylation-AUD associations because it is correlated with both sex and AUD status. Nevertheless, alcohol daily use amounts in control subjects (mean ± S.D.: 11 ± 9 gram) were much lower to that in AUD subjects (mean ± S.D.: 165 ± 81 gram) (Supplementary Table S6). By assessing alcohol daily use amounts, subjects were easily classified as AUD cases or controls. To circumvent the collinearity problem and avoid inaccurate coefficient estimation, we performed a case-control (or logistic regression) analysis rather than a linear regression analysis.

Second, we found that the majority of the differentially methylated CpGs identified in male AUD subjects were hypermethylated, and the hypermethylated CpGs were dispersed over different gene regions. Among the 1,201 hypermethylated CpGs (Fig. 1b), 417 (34.7%) were located in promoter regions, 503 (41.9%) were located in gene bodies, 82 (6.8%) were located in 3′ UTRs, and 199 (16.6%) were located in other regions. Our findings in promoter regions are consistent with those reported in most candidate gene studies that demonstrated increased DNA methylation levels in the promoter regions of AUD risk genes^5^,^6^,^7^,^8^,^9^,^10^,^11^. The association of promoter hypermethylation to risk of AUDs is relatively easier to understand because increased promoter DNA methylation may result in transcriptional silencing. Nevertheless, only about one-third of the hypermethylated CpGs identified in the present study are located in promoter regions, and most of the hypermethylated CpGs are located in non-promoter regions. Our findings suggest that altered DNA methylation in non-promoter regions may modulate gene transcription and confer increased vulnerability to AUDs. There is evidence that methylation of non-promoter CpGs located in gene bodies, enhancers, and insulators may influence the binding and function of regulatory proteins^37^. Further studies are warranted to confirm these findings and to gain a better understanding of the function of DNA methylation in non-promoter regions.

Third, we did not observe a clear-cut relationship between DNA methylation and gene expression. By integrative analysis of DNA methylome and gene transcriptome data, we discovered that DNA methylation and gene expression changes in the PFC of male AUD subjects were either positively or negatively correlated (Table 3). Besides 59 positively correlated CpG methylation-gene expression pairs (58 hypermethylated/upregulated and 1 hypomethylated/downregulated pairs), we also observed 47 negatively correlated CpG methylation-gene expression pairs (31 hypermethylated/downregulated and 16 hypomethylated/upregulated pairs). Considering that DNA methylation in promoter or transcription start site (TSS) regions may inhibit transcription initiation whereas DNA methylation in gene bodies may enhance transcription elongation^37^, we expect to observe a negative correlation between promoter DNA methylation and gene expression but a positive correlation between gene body DNA methylation and gene expression. However, the results from our study did not show such a simple relationship. The complex DNA methylation-gene expression relationship we observed in the present study may be due to at least two possible mechanisms. One possibility is that the influence of genetic variants, i.e, SNPs, on DNA methylation and/or gene expression potentially confounds the relationship between DNA methylation and gene expression. Our recent studies demonstrated that promoter DNA methylation levels of a number of genes involved in AUDs were significantly affected by genetic variants^38^. Thus, the effect of DNA methylation on gene transcription depends at least partially on the existence of underlying DNA sequence variants. Another possibility is that the gene-gene interaction network leads to a complex relationship between DNA methylation and gene expression. Because the CpG methylation-gene expression pairs obtained from the present study were generated by statistical (correlation) analyses, they do not reflect a direct biological relationship between DNA methylation and gene expression. Those genes involved in CpG methylation-gene expression pairs may be indirectly regulated by DNA methylation that impacts the transcription of other genes in the gene-gene interaction network. Of the 1,812 top CpGs identified in male AUD subjects, 611 were located in promoter regions. Except 41 promoter CpGs (29 hypermethylated and 12 hypomethylated) (Table 3), the remaining promoter CpGs did not show a significant contribution to their host gene transcription. A tentative explanation for this observation is that these promoter CpGs may not be located in transcription factor binding sites (TFBSs) or there are no interactive effects of the methylation of these promoter CpGs and the modification of histone proteins on gene transcription. Additionally, genetic variants around these promoter CpGs may influence their regulatory effects on gene transcription. Therefore, more effort is required to understand the function of DNA methylation on gene transcription.

Fourth, we found that some of the differentially methylated genes expressed in the PFC of male AUD subjects were enriched in GWAS-identified genes with variants associated with four types of substance dependencies (including alcohol dependence) or five psychiatric disorders. Our study indirectly demonstrated that some of the differentially methylated genes were potential genetic risk factors for AUDs. The enrichment analysis approach enabled us to appreciate the implications of the differentially methylated genes expressed in the PFC of AUD subjects. Our recent studies demonstrated that DNA methylation alterations in AUD subjects may be attributable to either genetic variants (also referred to as methylation quantitative trait loci or mQTLs)^38^ or non-genetic factors such as childhood adversity^39^ or chronic alcohol consumption^11^,^13^. In other words, the differentially methylated genes identified in AUD subjects may be simply biomarkers correlated to risk of AUDs, rather than causal factors that directly drive the occurrence of AUDs. Thus, there is a need to know which of the differentially methylated genes truly participate in the AUD pathway. Different complex disorders (particularly drug addiction and psychiatric disorders) may share genetic risk factors (i.e., pleiotropy)^40^. By enrichment analysis, we found that 36.4–56.7% of genes included in the turquoise module (M_turquoise_) and 32.4–55.6% of genes included in the blue module (M_blue_) were enriched in GWAS-identified genes containing variants associated with alcohol or drug dependence, or five specific psychiatric disorders. Similar enrichment patterns were observed when enrichment analysis was limited to genes expressed in the same brain region (i.e., the PFC) (Table 2). Despite the pleiotropic effects of a number of leading genes, other leading genes were heterogeneous with respect to effects for the four types of addiction and the five different psychiatric disorders. This may be due to the fact that different addiction and psychiatric disorders also have their own susceptibility and pathway genes. It can be reasonably speculated that the top differentially methylated genes (especially the leading genes identified in the enrichment analysis) could also serve as a prior resource to prioritize GWAS signals for addiction and other psychiatric disorders.

Several limitations of the present study must be overcome in future studies. First, although the size of our brain tissue sample was larger than that of any published studies, the statistical power of the sample was nevertheless limited. Thus, our findings should be replicated and extended in a larger postmortem collection of PFC samples. Second, the present study examined DNA methylation alterations in only one reward-related brain region (i.e., the PFC) of AUD subjects. Chronic alcohol exposure causes structural alteration of dendrites and their spines in multiple brain reward regions^41^, and DNA methylation is tissue-specific^42^,^43^. Hence, AUD-associated DNA methylation changes should be examined in additional brain regions such as the nucleus accumbens and the ventral tegmental area. Third, using postmortem samples, we cannot determine whether the observed DNA methylation and gene expression changes in AUD subjects occurred before (presumably caused by other environmental factors) or after heavy alcohol consumption. That is, all we can state is that there is an association – we cannot draw clear conclusions about cause or effect. To address this issue, we could use the dinking-in-the-dark mouse model that was applied in our recent study for analyzing alcohol drinking-induced DNA methylation alterations^13^.

In summary, this study demonstrated genome-wide DNA methylation changes in postmortem PFC of AUD subjects. These findings could increase our understanding of the biology of AUDs and the role of epigenetics in risk for this trait, and help in the development of pharmacotherapies for AUDs by targeting the differentially methylated genes or biological pathways identified here.

## Methods

### Postmortem PFC tissues

Autopsy brain tissue samples were obtained from the New South Wales Tissue Resource Centre (NSW TRC) at the University of Sydney. The NSW TRC is partially sponsored by the National Institute on Alcohol Abuse and Alcoholism (NIAAA) for collecting postmortem human brain tissues for alcohol-related research. It has ethics approval from the Sydney Local Health Network and The University of Sydney. Fresh-frozen sections of Brodmann area 9 (BA9, mainly the dorsolateral PFC) were obtained from 16 male and seven female pairs of AUD and control subjects (46 European Australians in total). Each pairs of AUD and control subjects were matched by sex, age, brain weight, brain pH, and postmortem interval (PMI). AUD subjects were affected with AUDs but not with illegal drugs of abuse or major psychotic disorders (such as schizophrenia and bipolar disorder) according to the criteria in the Diagnostic and Statistical Manual of Mental Disorders 4^th^ Edition (DSM-IV)^44^. Control subjects were assessed to be free of alcohol or drug abuse or dependence or major psychotic disorders. The sample information on AUD status, sex, age, alcohol daily use, PMI, brain weight, and brain pH is summarized in Supplementary Table S6.

### Genome-wide DNA methylation profiling

Genomic DNA was extracted from postmortem PFC tissues of 23 AUD and 23 control subjects using the QIAamp DNA Micro Kit (QIAGEN, Valencia, CA, USA). Genomic DNA was checked for quality by electrophoresis on a 0.7% agarose gel and quantified using a NanoDrop 8000 spectrophotometer (Thermo Scientific, Wilmington, DE, USA). 500 ng of genomic DNA was treated with bisulfite reagents included in the EZ-96 DNA methylation kit (Zymo Research, Orange, CA, USA) according to the manufacturer’s protocol. DNA methylation assays were performed at the Yale Center for Genome Analysis (YCGA). Genome-wide DNA methylation levels were examined using the Illumina Infinium HumanMethylation450 BeadChip (Illumina, San Diego, CA, USA), following the Illumina Infinium HD Methylation protocol^45^. Four chips (12 samples per chip) were used. Paired case and control samples were processed together on the same chip to reduce the impact of batch effects on microarray data. The BeadChip interrogates 485,577 CpG sites per sample at single-nucleotide resolution. It covers 99% of RefSeq genes, with 41% of CpG sites in promoter regions [from 1,500 bp upstream to 200 bp downstream of the transcription start site (TSS), including all or part of the 5′ untranslated region (5′ UTR) and/or the first exon], 31% in gene bodies, 3% in 3′ UTRs, and 25% in intergenic regions^45^. GenomeStudio software V2011.1 (Methylation Module V1.9.0) from Illumina was used to generate raw beta (β) values for each CpG site, with β values ranging from 0% (completely unmethylated) to 100% (completely methylated). The raw DNA methylation data was preprocessed by normalizing internal control probes that were designed specifically for the HumanMethylation450 BeadChip. Probes with intensities indistinguishable from background noises (detection *P*-value > 0.05) in any subject were excluded. Data from ch- probes (or non-CpG probes), SNP-CpG probes, cross-reactive probes were also excluded. The influence of two color channels and two types of CpG probes on CpG methylation measurements were eliminated using the R package *limma*^46^, a beta-mixture quantile normalization method (BMIQ)^47^ (Supplementary Fig. S3,S4). The batch effect (caused by the use of multiple chips in the assays) was compensated by normalizing the DNA methylation data using the ComBat function built-in R package *sva*^48^. Our DNA methylation data have been deposited in the NCBI GEO database (Accession Number: GSE49393).

After the above data preprocessing steps, 434,015 CpGs with high-quality methylation data were remained for analysis of differential DNA methylation in AUD subjects. Of the 434,015 CpGs, 34,840 CpGs (8.0%) were hypomethylated (β ≤ 0.2), 165,323 CpGs (38.1%) were hypermethylated (β ≥ 0.8), and the remaining CpGs were intermediately methylated (0.2 < β < 0.8). Density plotting of methylation levels of CpGs across the genomes of all 46 subjects in terms of their genome locations indicated that a high density of hypomethylated CpGs were located in CpG islands (CGIs) or gene promoter regions (Supplementary Fig. S5).

To assess the reproducibility of the array-based DNA methylation assay, the DNA methylome of a selected sample was profiled by three different chips. As shown in Supplementary Fig. S6a–c, the array-based DNA methylation assay was highly reproducible. Methylation levels of six CpGs in six genes (*AGT* cg01083716, *ALDH1L1* cg23141914, *GABRA1* cg02469186, *GRIN2C* cg16086007, *PAX6* cg09656389, and *SLC1A3* cg15841415), which showed differential methylation in AUD subjects by paired t-tests, were also determined by bisulfite sequencing^49^. *GRIN2C* cg16086007 and *AGT* cg01083716 failed this assay because no specific PCR products were obtained. As shown in Supplementary Fig. S6d, mean methylation levels of the other four CpGs measured by the two methods were highly correlated. Information regarding PCR primers and PCR conditions for bisulfite sequencing is provided in Supplementary Table S7.

### Differential DNA methylation analysis

Statistical analysis was carried out using the R package version 3.1.1. Differential DNA methylation was identified following two steps. In the first step, normalized methylation levels of 434,015 CpGs (those that remained after DNA methylation data quality managements) were marginally regressed on three covariates, i.e., sex, age, and PMI. In the second step, the residuals obtained from the first step were used to analyze DNA methylation differences between AUD cases and healthy controls using the empirical Bayesian linear model built-in R package *limma*^46^, which is a suitable tool for a small scale microarray study. The *q*-value was computed for each nominal *P* value by controlling the False Discovery Rate (FDR) at 0.05 using the *R* package *q-value*^50^. Considering the sex effect on DNA methylation^36^,^51^,^52^, differential DNA methylation analysis was conducted in men (16 male AUD cases *vs*. 16 matched male controls) and women (7 female AUD cases *vs*. 7 female matched controls). Differentially methylated CpGs in male or female AUD subjects were identified.

### Co-methylation network analysis

To assess the inter-correlation among CpGs, the co-methylation analysis was performed using the WGCNA R package^22^. The overall methylation level of CpGs clustered in a module was represented by the module eigengene (ME)^53^, which was equivalent to the first principal component (PC1). AUD-associated CpG clusters (or modules), in which CpGs were highly correlated in their methylation levels, were identified. For each module, the correlation of two parameters [the gene significance (GS) and the module membership (MM)] was evaluated. The GS stood for the magnitude of correlation between methylation levels of individual CpGs in the module and AUDs, and the MM meant the magnitude of correlation between methylation levels of individual CpGs in the module and the ME of the module. A significant correlation of GS and ME suggested that the differentially methylated CpGs identified in AUD subjects were also the most important (or central) elements of the module for AUDs. The hypergeometric-based test was also conducted to examine whether differentially methylated CpGs (*P*_nominal_ ≤ 0.05 and q ≤ 0.05) in AUD subjects were enriched in clustered CpGs included in AUD-associated CpG modules.

### Functional annotation by database DAVID

The function of genes mapped by differentially methylation CpGs that were also clustered in AUD-associated modules was annotated by the Database for Annotation, Visualization and Integrated Discovery (DAVID)^23^. The significance of gene ontology (GO) term enrichment was calculated according to a modified Fisher exact test (EASE < 0.1), and the top 10 GO terms together with fold enrichments were obtained from DAVID.

### Gene set enrichment analysis (GSEA) using genome-wide association study (GWAS) data

The GSEA method^54^,^55^ was used to assess whether genes mapped by differentially methylated CpGs included in AUD-associated CpG modules were over-represented by GWAS-identified genes with single nucleotide polymorphisms (SNPs) associated with alcohol dependence (AD)^24^, cocaine dependence (CD)^25^, opioid dependence (OD)^26^, or nicotine dependence (ND)^27^; the GSEA analysis was repeated in both African Americans and European Americans. Considering the genetic pleiotropy between substance dependence and psychiatric disorders, we also performed GSEA using GWAS data from the Psychiatric Genomics Consortium (PGC)^56^ for five psychiatric disorders such as attention deficit hyperactivity disorder (ADHD), autism spectrum disorder (ASD), bipolar disorder (BPD), major depressive disorder (MDD), and schizophrenia (SCZ); those GWAS data was described in published PGC studies^28^,^29^,^30^,^31^,^32^, and the summary statistics were obtained through public database (http://www.med.unc.edu/pgc/downloads). To generate the ranked gene list for GSEA, the genomic coordinates (i.e., the gene start and stop positions) of genes were first retrieved from the UCSC Genome Browser (data version hg19/GRCH 37). An intersection of 1,196,033 SNPs among the above 13 GWAS data was cross-checked and prepared before GSEA. For each GWAS data, *P* values of genes across the genome were assigned by minimal *P* values of CpGs or SNPs that were located from 10 kb upstream to 10 kb downstream of the gene, and the obtained *P* values of genes were ranked by −log_10_(*P* value). We also performed GSEA using only those genes that were found to be expressed in the PFC of human subjects in our recent study^35^, considering that drug addiction and psychiatric disorders are most often influenced by genes mainly expressed in the brain.

### Correlation analysis of DNA methylation and gene expression changes

We explored whether methylation levels of genes mapped by differentially methylated CpGs in postmortem PFC of AUD subjects were correlated with expression levels of differentially expressed gene in postmortem PFC of AUD subjects. Postmortem PFC genome-wide expression data for the same 16 pairs of male subjects (for the present study) were extracted from our previous study^35^. By co-expression analysis *via* WGCNA, 1,595 differentially expressed genes (*P*_nominal_ < 0.05) were clustered into five AUD-associated modules [E_turquoise_ (593 genes): *P* = 1.0 × 10^−3^); E_brown_ (234 genes): *P* = 1.0 × 10^−3^); E_blue_(575 genes): *P* = 1.0 × 10^−3^); E_yellow_(62 genes): *P* = 1.0 × 10^−3^; and E_grey_(n = 131 genes): *P* = 1.0 × 10^−13^]. We first examined pair-wise correlations between the overall methylation level (i.e., ME) of AUD-associated CpG modules and the overall gene expression level (i.e., ME) of the above five gene expression modules. We then analyzed the correlation of AUD-associated CpG methylation-gene expression pairs.

## Additional Information

**How to cite this article**: Wang, F. *et al*. DNA co-methylation modules in postmortem prefrontal cortex tissues of European Australians with alcohol use disorders. *Sci. Rep.* **6**, 19430; doi: 10.1038/srep19430 (2016).

## Supplementary Material

Supplementary Information

## Figures and Tables

**Table 1:** Top 20 hits identified in 32 male subjects (16 AUD cases *vs*. 16 controls).

CpGs	Chr.	Position	Genes	Location^a^	β^b^	Effect^c^	*P*_nominal_^d^	q^e^
cg19099433	2	3748379	*ALLC*	Body	0.92	−0.03	9.5 × 10^−9^	0.002
cg16460727	5	175297349	*CPLX2*	Promoter	0.87	−0.04	2.9 × 10^−7^	0.035
cg11938646	17	27050355	*SNORD42A*	Promoter	0.77	0.07	7.0 × 10^−7^	0.038
cg10767665	19	44279027	*KCNN4*	Body	0.17	0.05	9.6 × 10^−7^	0.038
cg05764102	16	72991344	*ZFHX3*	Promoter	0.91	−0.03	1.8 × 10^−6^	0.038
cg11562309	7	94286473	*PEG10*	Promoter	0.47	0.04	1.9 × 10^−6^	0.038
cg11415275	1	146715872	*CHD1L*	Body	0.13	0.05	2.6 × 10^−6^	0.038
cg09244748	11	63532530	*C11orf95*	Body	0.68	0.07	2.7 × 10^−6^	0.038
cg14250274	7	66206258	*RABGEF1*	Promoter	0.09	0.04	2.7 × 10^−6^	0.038
cg15836231	6	91490663		Intergenic	0.92	0.04	3.5 × 10^−6^	0.038
cg09249675	19	3401572	*NFIC*	Body	0.86	0.06	4.9 × 10^−6^	0.038
cg11344164	10	101878520		Intergenic	0.87	−0.05	5.5 × 10^−6^	0.038
cg14932133	2	121739247	*GLI2*	Body	0.90	−0.04	6.3 × 10^−6^	0.038
cg16695999	5	93905343	*C5orf36*	Promoter	0.81	0.05	6.5 × 10^−6^	0.038
cg17865555	19	49133647	*SPHK2*	3′UTR	0.26	0.10	7.0 × 10^−6^	0.038
cg13556266	4	8696173		Intergenic	0.83	−0.06	7.6 × 10^−6^	0.038
cg00147578	12	120730932		Intergenic	0.17	0.04	7.8 × 10^−6^	0.038
cg03064005	11	68142768	*LRP5*	Body	0.82	−0.07	8.3 × 10^−6^	0.039
cg07881365	19	15833929		Intergenic	0.29	0.08	8.8 × 10^−6^	0.039
cg06122518	1	168356673		Intergenic	0.20	0.06	9.4 × 10^−6^	0.039

^a^Location of CpGs in promoter regions, gene bodies, or 3′ untranslated regions (3′ UTRs).

^b^Average CpG methylation levels.

^c^Log-scale fold changes obtained by the R package *limma*.

^d^*P* values obtained by the R package *limma*.

^e^*q* values obtained by multiple testing controlling FDR at 0.05.

**Table 2:** Enrichment of genes mapped by CpGs in two large AUD-associated modules in GWAS-identified disease-associated genes.

	M_turquoise_ (n = 640)^a^			M_blue_ (n = 284)^a^			M_turquoise_ (n = 451)^b^			M_blue_ (n = 191)^b^		
	NES	n^c^	FDR q-val	NES	n^c^	FDR q-val	NES	n^c^	FDR q-val	NES	n^c^	FDR q-val
AD (AA)	1.49	297	0.006	1.25	158	0.027	1.47	213	0.013	1.07	69	0.287
CD (AA)	1.49	325	0.002	1.46	114	0.002	1.50	193	0.008	1.40	60	0.013
OD (AA)	1.47	330	0.003	1.50	113	0.006	1.43	164	0.013	1.46	78	0.016
ND (AA)	1.42	330	0.010	1.37	146	0.006	1.43	243	0.017	1.27	100	0.034
AD (EA)	1.45	328	0.005	1.48	111	0.005	1.39	196	0.014	1.50	77	0.008
CD (EA)	1.45	278	0.005	1.48	92	0.005	1.36	194	0.038	1.34	61	0.024
OD (EA)	1.34	276	0.006	1.48	110	0.014	1.40	183	0.008	1.43	73	0.008
ND (EA)	1.36	249	0.013	1.43	112	0.009	1.34	178	0.028	1.44	60	0.019
ADHD	1.37	363	0.006	1.45	116	0.006	1.39	154	0.016	1.42	80	0.014
ASD	1.33	305	0.011	1.40	116	0.008	1.33	255	0.036	1.31	55	0.038
BPD	1.41	292	0.006	1.43	135	0.010	1.40	213	0.025	1.39	58	0.018
MDD	1.43	326	0.003	1.66	115	<0.001	1.39	239	0.014	1.56	76	0.005
SCZ	1.29	233	0.014	1.56	112	0.003	1.34	145	0.020	1.51	73	0.007

AD, alcohol dependence; CD, cocaine dependence; OD, opioid dependence; ND, nicotine dependence.

ADHD, attention deficit hyperactivity disorder; ASD, autism spectrum disorders; BPD, bipolar disorder; MDD, major depressive disorder; SCZ, schizophrenia.

AA, African American. EA, European American.

GWAS, genome-wide associated study; NES, normalized enrichment score; FDR q value, false discovery rate (FDR) statistic.

Subjects for studying five psychiatric disorders (ADHD, ASD, BPD, MDD, and SCZ) were reported of European ancestry, as described by the Psychiatric GWAS Consortium (PGC) (http://www.med.unc.edu/pgc/results).

^a^Gene set enrichment analysis (GSEA) was performed using GWAS-identified disease-associated genes for four substance dependence traits (AD, CD, OD, or ND) or five psychiatric disorders (ADHD, ASD, BPD, MDD, or SCZ).

^b^Gene set enrichment analysis (GSEA) was performed using GWAS-identified disease-associated genes for four substance dependence traits (AD, CD, OD, or ND) or five psychiatric disorders (ADHD, ASD, BPD, MDD, or SCZ) and these genes were found to be expressed in human prefrontal cortex (PFC) in our previous study^35^.

^c^Number of leading genes when NES reached maximum in the enrichment analysis.

**Table 3:** 106 CpG methylation-gene expression correlation pairs in males.

Correlation Methylation-Expression	Number of pairs	Number of CpGs/genes	CpG Location			CpG Island	
			Promoter	Gene body	3′UTR	Yes	No
Hypermethylated/Upregulated	58	58/49	17	30	11	43	15
Hypermethylated/Downregulated	31	31/28	12	18	1	21	10
Hypomethylated/Upregulated	16	16/15	11	5	0	11	5
Hypomethylated/Downregulated	1	1/1	1	0	0	0	0
