# Supplementary material for: DNA co-methylation modules in postmortem prefrontal cortex tissues of European Australians with alcohol use disorders
Source: Sci Rep. 2016 Jan 14;6:19430. doi: 10.1038/srep19430 (PMC4725922; doi:10.1038/srep19430)
Supplement: Supplementary Information [file srep19430-s1.doc]

**DNA co-methylation modules in postmortem prefrontal cortex tissues of**

**European Australians with alcohol use disorders**

(Supplementary information)

Fan Wang, Hongqin Xu, Hongyu Zhao, Joel Gelernter, and Huiping Zhang

**Table S1.**  Biological processes enriched by top CpGs in two AUD-associated modules.

**Table S2.** Pair-wise correlation of AUD-associated CpG methylation modules and AUD-associated gene expression modules.

**Table S3.** Differentially expression genes paired with hypomethylated CpGs.

**Table S4.** Differentially expression genes paired with hypomethylated CpGs.

**Table S5.**  Biological processes enriched by genes involved in 106 CpG methylation-gene expression pairs.

**Table S6.** Clinical characteristics of 46 postmortem prefrontal cortex (PFC) tissue samples.

**Table S7.**  Primers designed for measuring methylation levels of six CpGs by bisulfite Sanger sequencing.

**Table S8.** Top 10 pathways enriched by 104 genes mapped by the top 154 differentially methylated CpGs identified in female AUD subjects

**Figure S1.** Differentially methylated CpGs in all or female subjects with alcohol use disorders (AUDs).

**Figure S2.** Co-methylation analysis of nominally significant CpGs identified in male subjects with alcohol use disorders (AUDs).

**Figure S3.** Distribution of CpG levels determined by two color channels before (a) and after (b) adjusted by the R package *lumi*.

**Figure S4.**  Distribution of CpG methylation levels determined by two types of probes across all 46 samples.

**Figure S5.** Distributions of methylation levels of 434,015 CpGs across the genome.

**Figure S6.** Assessing reproducibility of Illumina Infinium HumanMethylation450 BeadChip assays and validating methylation levels of six CpGs using bisulfite Sanger sequencing.

**Table S1. Biological processes enriched by top CpGs in two AUD-associated modules.**

| GO ID | GO Term | Count | Fold Enrichment | *P* value | *P*_Benjamini_ | Genes |
| --- | --- | --- | --- | --- | --- | --- |
| ***Module (turquoise)*** | | | | | | |
| GO:0030030 | cell projection organization | 33 | 2.5 | 3.7E-06 | 9.4E-03 | *PARD3, NRP1, PDGFA, PAX6, MTSS1L, LPAR1, EPHB3, PRKG1, GLI2, MYCBP2, AKT1, ATP2B2, C19ORF20, BAI1, RTN4RL2, SEMA3B, DCLK1, EGFR, PRKCA, KLF7, STMN3, PLEK, BAIAP2, RXRA, SPTBN4, NOTCH1, ITGA6, MAP1S, ULK1, LAMA5, MAPK8IP3, APBB2, MYH10* |
| GO:0048858 | cell projection morphogenesis | 23 | 2.6 | 7.7E-05 | 2.7E-02 | *PRKCA, EGFR, KLF7, PARD3, NRP1, BAIAP2, RXRA, SPTBN4, PAX6, EPHB3, GLI2, MYCBP2, NOTCH1, MAP1S, ULK1, LAMA5, BAI1, MAPK8IP3, SEMA3B, RTN4RL2, APBB2, DCLK1, MYH10* |
| GO:0000904 | cell morphogenesis involved in differentiation | 23 | 2.6 | 7.2E-05 | 3.0E-02 | *PRKCA, KLF7, PARD3, NRP1, BAIAP2, RXRA, SPTBN4, PAX6, GLI2, SOX9, EPHB3, MYCBP2, ATP2B2, NOTCH1, ULK1, LAMA5, BAI1, MAPK8IP3, SEMA3B, RTN4RL2, APBB2, DCLK1, MYH10* |
| GO:0048666 | neuron development | 28 | 2.3 | 9.8E-05 | 3.1E-02 | *PARD3, NRP1, OPCML, PAX6, GLI2, EPHB3, PRKG1, MYCBP2, ATP2B2, BAI1, RTN4RL2, SEMA3B, DCLK1, EGFR, PRKCA, KLF7, STMN3, RXRA, BAIAP2, SPTBN4, NOTCH1, MAP1S, ULK1, VEGFA, MAPK8IP3, APBB2, NTM, MYH10* |
| GO:0051056 | regulation of small GTPase mediated signal transduction | 23 | 2.5 | 1.2E-04 | 3.2E-02 | *ARHGEF4, OBSCN, ABR, AGFG1, RAP1GAP, STMN3, CYTH1, ARHGEF7, ASAP2, TRIO, CDH2, LPAR1, MCF2L, TIAM2, DGKZ, MLST8, AGAP1, SYNGAP1, RASA3, KNDC1, TBC1D20, ARAP1, IQSEC1* |
| GO:0007409 | axonogenesis | 20 | 2.9 | 7.0E-05 | 3.5E-02 | *PRKCA, KLF7, PARD3, NRP1, RXRA, BAIAP2, SPTBN4, PAX6, EPHB3, GLI2, MYCBP2, NOTCH1, ULK1, BAI1, MAPK8IP3, SEMA3B, RTN4RL2, APBB2, DCLK1, MYH10* |
| GO:0048812 | neuron projection morphogenesis | 22 | 2.9 | 2.9E-05 | 3.6E-02 | *PRKCA, EGFR, KLF7, PARD3, NRP1, RXRA, BAIAP2, SPTBN4, PAX6, EPHB3, GLI2, MYCBP2, NOTCH1, MAP1S, ULK1, BAI1, MAPK8IP3, SEMA3B, RTN4RL2, APBB2, DCLK1, MYH10* |
| GO:0032990 | cell part morphogenesis | 23 | 2.5 | 1.5E-04 | 3.6E-02 | *PRKCA, EGFR, KLF7, PARD3, NRP1, BAIAP2, RXRA, SPTBN4, PAX6, EPHB3, GLI2, MYCBP2, NOTCH1, MAP1S, ULK1, LAMA5, BAI1, MAPK8IP3, SEMA3B, RTN4RL2, APBB2, DCLK1, MYH10* |
| GO:0048667 | cell morphogenesis involved in neuron differentiation | 21 | 2.8 | 6.7E-05 | 4.2E-02 | *PRKCA, KLF7, PARD3, NRP1, RXRA, BAIAP2, SPTBN4, PAX6, EPHB3, GLI2, MYCBP2, ATP2B2, NOTCH1, ULK1, BAI1, MAPK8IP3, SEMA3B, RTN4RL2, APBB2, DCLK1, MYH10* |
| GO:0031175 | neuron projection development | 24 | 2.6 | 5.3E-05 | 4.4E-02 | *PRKCA, EGFR, KLF7, PARD3, NRP1, STMN3, BAIAP2, RXRA, SPTBN4, PAX6, GLI2, PRKG1, EPHB3, MYCBP2, NOTCH1, MAP1S, ULK1, BAI1, MAPK8IP3, SEMA3B, RTN4RL2, APBB2, DCLK1, MYH10* |
| ***Module (blue)*** | | | | | | |
| GO:0048511 | rhythmic process | 9 | 4.6 | 7.1E-04 | 6.5E-01 | *TAF4, CRY2, PTGDS, BCL2, SLC9A3, ARNTL, BMPR1B, NFIL3, ENOX1* |
| GO:0045893 | positive regulation of transcription, DNA-dependent | 17 | 2.4 | 2.4E-03 | 8.3E-01 | *SREBF1, HNF1B, ELF1, CAMTA2, THRA, ESRRG, ARNTL, ZEB1, SOX9, SEC14L2, HMGA1, PLAGL1, NOTCH1, HES5, ZMIZ1, MAPRE3, ARHGEF10L* |
| GO:0010604 | positive regulation of macromolecule metabolic process | 25 | 1.9 | 2.5E-03 | 7.1E-01 | *HNF1B, ELF1, CAMTA2, THRA, ZEB1, SOX9, IL34, SEC14L2, PLAGL1, BCL2, C1QTNF2, PSMD7, ARHGEF10L, SREBF1, LYN, ESRRG, TOPORS, ARNTL, HMGA1, NOTCH1, PIAS4, HES5, ZMIZ1, TNK2, MAPRE3* |
| GO:0051254 | positive regulation of RNA metabolic process | 17 | 2.3 | 2.6E-03 | 6.2E-01 | *SREBF1, HNF1B, ELF1, CAMTA2, THRA, ESRRG, ARNTL, ZEB1, SOX9, SEC14L2, HMGA1, PLAGL1, NOTCH1, HES5, ZMIZ1, MAPRE3, ARHGEF10L* |
| GO:0006357 | regulation of transcription from RNA polymerase II promoter | 22 | 2.0 | 3.2E-03 | 6.2E-01 | *SREBF1, ERF, HNF1B, TAF4, ELF1, CAMTA2, THRA, CBX2, ZEB1, ARNTL, SOX9, PLAGL1, HOXB4, NOTCH1, PIAS4, HES5, MED16, ZMIZ1, JAZF1, ARHGEF10L, KDM6B, NCOR2* |
| GO:0045941 | positive regulation of transcription | 18 | 2.1 | 5.2E-03 | 7.2E-01 | *SREBF1, HNF1B, ELF1, CAMTA2, THRA, ESRRG, TOPORS, ARNTL, ZEB1, SOX9, SEC14L2, HMGA1, PLAGL1, NOTCH1, HES5, ZMIZ1, MAPRE3, ARHGEF10L* |
| GO:0051789 | response to protein stimulus | 7 | 4.3 | 5.6E-03 | 6.9E-01 | *CD48, LYN, BCL2, ATF6B, AMFR, DNAJB1, DDAH2* |
| GO:0010628 | positive regulation of gene expression | 18 | 2.0 | 6.9E-03 | 7.2E-01 | *SREBF1, HNF1B, ELF1, CAMTA2, THRA, ESRRG, TOPORS, ARNTL, ZEB1, SOX9, SEC14L2, HMGA1, PLAGL1, NOTCH1, HES5, ZMIZ1, MAPRE3, ARHGEF10L* |
| GO:0009891 | positive regulation of biosynthetic process | 20 | 1.9 | 8.9E-03 | 7.7E-01 | *SREBF1, HNF1B, ELF1, CAMTA2, THRA, ESRRG, TOPORS, ARNTL, ZEB1, SOX9, ABCG4, SEC14L2, HMGA1, PLAGL1, NOTCH1, HES5, ZMIZ1, C1QTNF2, MAPRE3, ARHGEF10L* |
| GO:0010557 | positive regulation of macromolecule biosynthetic process | 19 | 1.9 | 1.0E-02 | 7.8E-01 | *SREBF1, HNF1B, ELF1, CAMTA2, THRA, ESRRG, TOPORS, ARNTL, ZEB1, SOX9, SEC14L2, HMGA1, PLAGL1, NOTCH1, HES5, ZMIZ1, C1QTNF2, MAPRE3, ARHGEF10L* |

**Table S2.** Pair-wise correlation of AUD-associated CpG methylation modules and AUD-associated gene expression modules.

| 22 CpG  modules | CpGs | Five AUD-associated gene expression modules | | | | |
| --- | --- | --- | --- | --- | --- | --- |
|  |  | E_turquoise_  (n=593 ) | E_brown_  (n=234) | E_blue_  (n=575) | E_yellow_  (n=62) | E_grey_  (n=131) |
| M_lightyellow_ | 95 | **-0.34 (2)** | 0.27 (1) | **0.36 (2)** | **0.36 (1)** | **-0.67 (0)** |
| M_black_ | 373 | -0.26 (10) | **0.41 (1)** | 0.16 (8) | **0.38 (1)** | **-0.68 (2)** |
| M_blue_ | 18,061 | **-0.52 (226)** | **0.51 (58)** | **0.42 (229)** | **0.46 (27)** | **-0.56 (32)** |
| M_lightgreen_ | 111 | **-0.47 (1)** | **0.61 (0)** | **0.38 (3)** | 0.25 (0) | **-0.55 (0)** |
| M_pink_ | 289 | **-0.53 (4)** | **0.48 (0)** | **0.53 (7)** | **0.41 (2)** | **-0.63 (2)** |
| M_cyan_ | 169 | **-0.53 (1)** | **0.55 (1)** | **0.43 (3)** | **0.39 (0)** | **-0.55 (1)** |
| M_greenyellow_ | 211 | **-0.47 (3)** | **0.49 (2)** | **0.5 (4)** | 0.19 (0) | **-0.58 (0)** |
| M_midnightblue_ | 164 | **-0.53 (2)** | **0.55 (1)** | **0.46 (3)** | 0.29 (0) | **-0.47 (0)** |
| M_turquoise_ | 42,389 | **-0.52 (319)** | 0.33 (102) | **0.52 (351)** | **0.39 (41)** | **-0.47 (51)** |
| M_purple_ | 229 | **-0.59 (3)** | **0.37 (2)** | **0.41 (4)** | **0.65 (1)** | **-0.59 (1)** |
| M_lightcyan_ | 161 | **-0.37 (7)** | **0.4 (1)** | 0.32 (0) | 0.3 (0) | **-0.68 (0)** |
| M_salmon_ | 198 | -0.31 (3) | **0.34 (2)** | 0.31 (2) | **0.36 (1)** | **-0.62 (1)** |
| M_darkred_ | 81 | **-0.39 (2)** | **0.43 (1)** | **0.35 (2)** | **0.29 (0)** | **-0.62 (1)** |
| M_magenta_ | 261 | **-0.52 (6)** | **0.54 (2)** | **0.43 (3)** | **0.49 (0)** | **-0.63 (1)** |
| M_green_ | 1,890 | -0.35 (53) | **0.51 (21)** | 0.25 (40) | 0.31 (2) | **-0.69 (9)** |
| M_grey60_ | 116 | -0.18 (3) | 0.26 (0) | 0.16 (5) | 0.25 (1) | **-0.61 (2)** |
| M_yellow_ | 3,312 | **0.35 (47)** | **-0.46 (7)** | -0.16 (51) | **-0.37 (10)** | **0.47 (5)** |
| M_red_ | 1,103 | **0.55 (8)** | **-0.53 (5)** | **-0.58 (16)** | -0.36 (0) | **0.47 (4)** |
| M_royalblue_ | 92 | **0.51 (4)** | **-0.5 (0)** | **-0.59 (3)** | -0.28 (0) | **0.54 (1)** |
| M_brown_ | 8,469 | **0.52 (112)** | **-0.38 (27)** | **-0.51 (138)** | **-0.37 (19)** | **0.49 (14)** |
| M_tan_ | 199 | 0.28 (3) | -0.17 (3) | **-0.49 (5)** | -0.17 (0) | **0.59 (0)** |
| M_grey_ | 9,615 | **-0.53 (183)** | **0.58 (55)** | **0.51 (157)** | **0.47 (16)** | **-0.9 (27)** |

The numbers outside the brackets were correlation coefficients, and the numbers inside the brackets were overlapped genes. Significant correlations (*P* < 0.05) were bold.

**Table S3.** Differentially expression genes paired with hypermethylated CpGs.

| CpGs | Chr. | Position | Genes | Locations^a^ | Module^b^ | DNA Methylation | | |  | Gene Expression | |
| --- | --- | --- | --- | --- | --- | --- | --- | --- | --- | --- | --- |
|  |  |  |  |  |  | β^c^ | Effect | *P*_nominal_ |  | Effect | *P*_nominal_ |
| cg09294095 | 11 | 131630464 | *NTM* | Body | blue | 0.18 | 0.06 | 3.5E-04 |  | 0.24 | 4.6E-04 |
| cg12079699 | 11 | 131564481 | *NTM* | Body | turquoise | 0.83 | 0.05 | 4.9E-04 |  | 0.24 | 4.6E-04 |
| cg08597733 | 20 | 60886158 | *LAMA5* | Body | turquoise | 0.88 | 0.06 | 5.1E-04 |  | 0.36 | 1.3E-03 |
| cg12390057 | 20 | 33585849 | *MYH7B* | Body | turquoise | 0.83 | 0.03 | 2.8E-04 |  | -0.14 | 2.3E-03 |
| cg18459342 | 8 | 81084056 | *TPD52* | Promoter | turquoise | 0.25 | 0.05 | 7.2E-04 |  | -0.10 | 2.4E-03 |
| cg24262376 | 1 | 151137274 | *SCNM1* | Promoter | blue | 0.46 | 0.06 | 4.4E-04 |  | -0.07 | 4.0E-03 |
| cg17057702 | 12 | 125262508 | *SCARB1* | 3'UTR | turquoise | 0.88 | 0.05 | 1.3E-04 |  | 0.15 | 4.4E-03 |
| cg09510128 | 7 | 1132036 | *GPER* | Body | turquoise | 0.79 | 0.09 | 7.0E-04 |  | 0.36 | 4.6E-03 |
| cg23707289 | 18 | 60988099 | *BCL2* | Promoter | blue | 0.12 | 0.05 | 5.6E-04 |  | 0.15 | 4.6E-03 |
| cg01720920 | 14 | 104185151 | *ZFYVE21* | Body | turquoise | 0.52 | 0.06 | 5.9E-04 |  | 0.26 | 4.9E-03 |
| cg18856091 | 2 | 105956343 | *C2orf49* | Body | blue | 0.81 | 0.06 | 1.1E-05 |  | -0.11 | 4.9E-03 |
| cg16668180 | 16 | 46963261 | *GPT2* | 3'UTR | turquoise | 0.70 | 0.07 | 3.3E-04 |  | 0.35 | 5.2E-03 |
| cg02106534 | 16 | 4561545 | *C16orf5* | 3'UTR | turquoise | 0.39 | 0.03 | 1.9E-04 |  | 0.12 | 5.3E-03 |
| cg10126715 | 1 | 37500195 | *GRIK3* | Promoter | turquoise | 0.30 | 0.03 | 3.8E-04 |  | -0.06 | 7.2E-03 |
| cg10206380 | 16 | 57672032 | *GPR56* | Promoter | turquoise | 0.61 | 0.12 | 5.5E-04 |  | 0.46 | 7.4E-03 |
| cg04145890 | 4 | 1007657 | *FGFRL1* | Body | blue | 0.66 | 0.05 | 3.8E-04 |  | 0.40 | 8.3E-03 |
| cg00119073 | 3 | 187870713 | *LPP* | Promoter | turquoise | 0.15 | 0.06 | 2.2E-04 |  | 0.34 | 8.5E-03 |
| cg01811796 | 1 | 26233565 | *STMN1* | Promoter | turquoise | 0.36 | 0.06 | 6.5E-04 |  | -0.21 | 8.6E-03 |
| cg20020161 | 2 | 231732669 | *ITM2C* | Body | turquoise | 0.46 | 0.06 | 3.3E-04 |  | 0.20 | 1.0E-02 |
| cg05681859 | 6 | 30698734 | *FLOT1* | Body | turquoise | 0.45 | 0.05 | 2.8E-04 |  | -0.11 | 1.0E-02 |
| cg04353171 | 6 | 30698729 | *FLOT1* | Body | brown | 0.50 | 0.05 | 3.4E-04 |  | -0.11 | 1.0E-02 |
| cg20821885 | 17 | 65689115 | *PITPNC1* | 3'UTR | grey | 0.89 | 0.05 | 1.2E-04 |  | 0.26 | 1.0E-02 |
| cg13119035 | 7 | 42951436 | *C7orf25* | Promoter | turquoise | 0.32 | 0.03 | 1.7E-05 |  | -0.14 | 1.1E-02 |
| cg01869554 | 15 | 90772557 | *SEMA4B* | 3'UTR | turquoise | 0.73 | 0.07 | 1.1E-04 |  | 0.20 | 1.5E-02 |
| cg24482286 | 1 | 150981652 | *FAM63A* | Promoter | turquoise | 0.09 | 0.04 | 6.3E-04 |  | 0.23 | 1.5E-02 |
| cg05216501 | 19 | 35632564 | *FXYD1* | Body | blue | 0.08 | 0.04 | 3.9E-04 |  | 0.26 | 1.6E-02 |
| cg09489844 | 17 | 79880647 | *MAFG* | Body | turquoise | 0.91 | 0.04 | 6.3E-04 |  | -0.12 | 1.8E-02 |
| cg01595717 | 9 | 140586201 | *EHMT1* | Body | turquoise | 0.27 | 0.10 | 3.3E-04 |  | 0.03 | 1.9E-02 |
| cg13791668 | 9 | 140568043 | *EHMT1* | Body | turquoise | 0.90 | 0.03 | 5.7E-04 |  | 0.03 | 1.9E-02 |
| cg04450857 | 10 | 119296756 | *EMX2OS* | Body | blue | 0.51 | 0.07 | 1.5E-04 |  | 0.30 | 1.9E-02 |
| cg26517831 | 7 | 2768566 | *GNA12* | 3'UTR | turquoise | 0.77 | 0.07 | 6.4E-04 |  | 0.16 | 1.9E-02 |
| cg03812676 | 11 | 67254381 | *AIP* | Body | brown | 0.87 | 0.04 | 3.9E-04 |  | -0.09 | 2.0E-02 |
| cg22888967 | 13 | 29293801 | *SLC46A3* | Promoter | blue | 0.47 | 0.04 | 3.6E-04 |  | 0.12 | 2.1E-02 |
| cg21901488 | 2 | 128412420 | *LIMS2* | Body | turquoise | 0.70 | 0.05 | 3.6E-04 |  | 0.18 | 2.1E-02 |
| cg12416929 | 6 | 108882205 | *FOXO3* | Promoter | turquoise | 0.23 | 0.05 | 1.1E-04 |  | 0.11 | 2.2E-02 |
| cg05958351 | 16 | 2804731 | *SRRM2* | Promoter | turquoise | 0.65 | 0.06 | 6.1E-04 |  | 0.11 | 2.3E-02 |
| cg18206768 | 7 | 2582681 | *C7orf27* | Body | turquoise | 0.84 | 0.06 | 7.0E-04 |  | 0.14 | 2.3E-02 |
| cg03917138 | 17 | 70120182 | *SOX9* | Body | turquoise | 0.33 | 0.14 | 3.5E-04 |  | 0.46 | 2.6E-02 |
| cg13058710 | 17 | 70119609 | *SOX9* | Body | blue | 0.20 | 0.07 | 3.9E-04 |  | 0.46 | 2.6E-02 |
| cg19705159 | 7 | 102109446 | *LRWD1* | Body | turquoise | 0.83 | 0.06 | 6.0E-04 |  | -0.09 | 2.6E-02 |
| cg20941739 | 11 | 133035052 | *OPCML* | Body | turquoise | 0.61 | 0.08 | 6.9E-04 |  | -0.19 | 2.6E-02 |
| cg04682699 | 3 | 50248845 | *SLC38A3* | Promoter | turquoise | 0.74 | 0.06 | 2.8E-04 |  | 0.15 | 2.6E-02 |
| cg19122460 | 3 | 50257857 | *SLC38A3* | 3'UTR | turquoise | 0.63 | 0.08 | 5.8E-04 |  | 0.15 | 2.6E-02 |
| cg03175771 | 16 | 30661534 | *PRR14* | Promoter | blue | 0.11 | 0.03 | 4.8E-04 |  | 0.13 | 2.7E-02 |
| cg09618385 | 16 | 87864826 | *SLC7A5* | 3'UTR | turquoise | 0.61 | 0.07 | 6.1E-04 |  | 0.27 | 2.8E-02 |
| cg23119809 | 17 | 72839750 | *GRIN2C* | Body | blue | 0.54 | 0.05 | 1.8E-05 |  | 0.46 | 2.8E-02 |
| cg12536809 | 17 | 72852514 | *GRIN2C* | Promoter | blue | 0.61 | 0.05 | 5.3E-04 |  | 0.46 | 2.8E-02 |
| cg15675740 | 11 | 45874617 | *CRY2* | Body | blue | 0.28 | 0.04 | 5.8E-05 |  | 0.17 | 2.8E-02 |
| cg08529333 | 2 | 224904837 | *SERPINE2* | Promoter | brown | 0.32 | 0.08 | 6.2E-04 |  | 0.26 | 2.9E-02 |
| cg05953751 | 17 | 80054291 | *FASN* | Body | turquoise | 0.56 | 0.05 | 5.4E-05 |  | -0.13 | 2.9E-02 |
| cg25068915 | 17 | 80052907 | *FASN* | Body | turquoise | 0.86 | 0.04 | 2.3E-04 |  | -0.13 | 2.9E-02 |
| cg14797580 | 17 | 80053635 | *FASN* | Body | turquoise | 0.67 | 0.06 | 3.3E-04 |  | -0.13 | 2.9E-02 |
| cg26657242 | 7 | 1488315 | *MICALL2* | Body | turquoise | 0.71 | 0.08 | 3.1E-04 |  | 0.06 | 2.9E-02 |
| cg04500377 | 8 | 38090208 | *DDHD2* | Promoter | turquoise | 0.24 | 0.04 | 5.5E-04 |  | -0.08 | 2.9E-02 |
| cg12305870 | 20 | 398182 | *RBCK1* | Body | blue | 0.78 | 0.03 | 3.2E-04 |  | 0.11 | 2.9E-02 |
| cg09713225 | 20 | 400412 | *RBCK1* | Body | turquoise | 0.76 | 0.11 | 3.9E-04 |  | 0.11 | 2.9E-02 |
| cg03414569 | 3 | 184971699 | *EHHADH* | Promoter | turquoise | 0.11 | 0.04 | 5.2E-04 |  | 0.11 | 3.1E-02 |
| cg04497094 | 17 | 57741573 | *CLTC* | Body | turquoise | 0.90 | 0.05 | 2.5E-04 |  | -0.12 | 3.2E-02 |
| cg27090007 | 13 | 28519388 | *ATP5EP2* | Body | turquoise | 0.18 | 0.04 | 6.1E-05 |  | -0.09 | 3.2E-02 |
| cg14129790 | 14 | 102510790 | *DYNC1H1* | Body | turquoise | 0.61 | 0.06 | 5.7E-04 |  | -0.10 | 3.2E-02 |
| cg14688588 | 3 | 184870705 | *C3orf70* | Promoter | brown | 0.29 | 0.04 | 1.3E-04 |  | 0.26 | 3.3E-02 |
| cg15673491 | 6 | 30885481 | *VARS2* | Body | turquoise | 0.74 | 0.05 | 5.4E-04 |  | 0.18 | 3.3E-02 |
| cg05623411 | 9 | 14691289 | *ZDHHC21* | Promoter | turquoise | 0.66 | 0.10 | 3.4E-04 |  | -0.07 | 3.4E-02 |
| cg07249860 | 13 | 36421844 | *DCLK1* | Body | turquoise | 0.91 | 0.05 | 2.2E-04 |  | -0.32 | 3.5E-02 |
| cg12278705 | 1 | 86045474 | *DDAH1* | Promoter | turquoise | 0.22 | 0.06 | 1.9E-04 |  | 0.16 | 3.5E-02 |
| cg05359983 | 17 | 48782688 | *ANKRD40* | Body | turquoise | 0.87 | 0.07 | 4.0E-05 |  | -0.15 | 3.7E-02 |
| cg19194237 | 2 | 73485189 | *FBXO41* | 3'UTR | turquoise | 0.60 | 0.04 | 7.0E-04 |  | 0.17 | 3.7E-02 |
| cg03239386 | 19 | 45999978 | *RTN2* | Body | magenta | 0.23 | 0.06 | 5.1E-04 |  | -0.15 | 3.8E-02 |
| cg19567168 | 12 | 6438502 | *TNFRSF1A* | Body | turquoise | 0.80 | 0.09 | 6.5E-04 |  | 0.29 | 3.8E-02 |
| cg10313633 | 11 | 44972890 | *TP53I11* | Promoter | blue | 0.63 | 0.06 | 2.0E-04 |  | 0.06 | 3.9E-02 |
| cg04084236 | 4 | 143766602 | *INPP4B* | Promoter | blue | 0.27 | 0.06 | 2.8E-04 |  | -0.09 | 3.9E-02 |
| cg24092907 | 7 | 12729367 | *ARL4A* | 3'UTR | turquoise | 0.73 | 0.06 | 4.1E-04 |  | 0.09 | 4.0E-02 |
| cg22512208 | 7 | 2566263 | *LFNG* | Body | turquoise | 0.89 | 0.05 | 4.7E-05 |  | 0.21 | 4.0E-02 |
| cg01069159 | 5 | 161275865 | *GABRA1* | Promoter | turquoise | 0.10 | 0.04 | 3.6E-04 |  | -0.33 | 4.1E-02 |
| cg04875697 | 9 | 137228542 | *RXRA* | Body | turquoise | 0.83 | 0.07 | 2.0E-04 |  | 0.16 | 4.2E-02 |
| cg13061648 | 9 | 137331317 | *RXRA* | 3'UTR | turquoise | 0.85 | 0.03 | 3.2E-04 |  | 0.16 | 4.2E-02 |
| cg13413384 | 9 | 137302231 | *RXRA* | Body | turquoise | 0.79 | 0.04 | 6.3E-04 |  | 0.16 | 4.2E-02 |
| cg05573019 | 5 | 54601993 | *DHX29* | Body | red | 0.90 | 0.03 | 4.0E-04 |  | -0.09 | 4.3E-02 |
| cg05062694 | 2 | 27342324 | *CGREF1* | Promoter | brown | 0.85 | 0.04 | 7.1E-04 |  | -0.06 | 4.4E-02 |
| cg20541656 | 8 | 98787334 | *LAPTM4B* | Promoter | turquoise | 0.15 | 0.05 | 4.3E-04 |  | -0.20 | 4.4E-02 |
| cg02461011 | 15 | 90777825 | *CIB1* | Promoter | turquoise | 0.13 | 0.05 | 6.4E-04 |  | 0.15 | 4.5E-02 |
| cg15212295 | 17 | 64710687 | *PRKCA* | Body | turquoise | 0.52 | 0.08 | 6.1E-04 |  | 0.15 | 4.7E-02 |
| cg13826564 | 11 | 65306731 | *LTBP3* | Body | turquoise | 0.52 | 0.09 | 6.6E-04 |  | 0.08 | 4.8E-02 |
| cg18045515 | 17 | 77751069 | *CBX2* | Promoter | blue | 0.35 | 0.11 | 4.7E-04 |  | 0.04 | 4.8E-02 |
| cg01347335 | 3 | 10491630 | *ATP2B2* | Promoter | turquoise | 0.82 | 0.04 | 6.0E-04 |  | -0.20 | 4.8E-02 |
| cg03228209 | 8 | 145814162 | *KIAA1688* | Body | brown | 0.62 | 0.04 | 2.7E-04 |  | 0.15 | 4.9E-02 |
| cg04277232 | 6 | 31512618 | *ATP6V1G2* | 3'UTR | blue | 0.44 | 0.05 | 2.3E-04 |  | -0.18 | 4.9E-02 |
| cg26978172 | 6 | 31696223 | *DDAH2* | Body | blue | 0.58 | 0.11 | 3.3E-04 |  | 0.17 | 5.0E-02 |
| cg23348155 | 6 | 31696319 | *DDAH2* | Body | blue | 0.59 | 0.04 | 7.2E-04 |  | 0.17 | 5.0E-02 |

^a^ Location of CpGs in promoter regions, gene bodies, or 3' untranslated regions (3' UTRs).

^b^ Module membership by co-methylation analysis.

^c^ Average CpG methylation levels.

**Table S4.** Differentially expression genes paired with hypomethylated CpGs.

| CpGs | Chr. | Position | Genes | Location^a^ | Module^b^ | DNA Methylation | | |  | Gene Expression | |
| --- | --- | --- | --- | --- | --- | --- | --- | --- | --- | --- | --- |
|  |  |  |  |  |  | β^c^ | Effect | *P*_nominal_ |  | Effect | *P*_nominal_ |
| cg22401505 | 6 | 116893550 | *RWDD1* | Promoter | turquoise | 0.45 | -0.05 | 4.8E-04 |  | 0.10 | 5.0E-03 |
| cg01347228 | 17 | 29297391 | *RNF135* | Promoter | turquoise | 0.81 | -0.05 | 4.6E-04 |  | 0.16 | 5.8E-03 |
| cg11412288 | 6 | 117802725 | *DCBLD1* | Promoter | brown | 0.73 | -0.09 | 3.0E-04 |  | 0.18 | 6.7E-03 |
| cg09421562 | 17 | 56357994 | *MPO* | Promoter | turquoise | 0.85 | -0.04 | 7.2E-04 |  | 0.18 | 6.7E-03 |
| cg19584674 | 14 | 24898818 | *CBLN3* | Promoter | blue | 0.47 | -0.08 | 5.3E-04 |  | 0.26 | 6.8E-03 |
| cg10854441 | 22 | 50524691 | *MLC1* | Promoter | blue | 0.26 | -0.06 | 3.8E-04 |  | 0.50 | 1.1E-02 |
| cg09061733 | 11 | 57364841 | *SERPING1* | Promoter | yellow | 0.30 | -0.09 | 5.9E-04 |  | 0.24 | 1.3E-02 |
| cg05939495 | 15 | 74660110 | *CYP11A1* | Promoter | brown | 0.11 | -0.03 | 1.4E-04 |  | 0.32 | 1.6E-02 |
| cg11229284 | 3 | 122399506 | *PARP14* | Promoter | brown | 0.51 | -0.03 | 2.2E-04 |  | 0.27 | 1.7E-02 |
| cg09780231 | 17 | 873452 | *NXN* | Body | blue | 0.37 | -0.10 | 3.6E-04 |  | 0.05 | 1.7E-02 |
| cg18513062 | 17 | 763049 | *NXN* | Body | brown | 0.73 | -0.09 | 1.3E-04 |  | 0.16 | 2.2E-02 |
| cg07449753 | 7 | 83057120 | *SEMA3E* | Body | turquoise | 0.85 | -0.07 | 5.6E-04 |  | 0.19 | 2.7E-02 |
| cg21764190 | 11 | 31835534 | *PAX6* | Promoter | turquoise | 0.89 | -0.03 | 2.6E-04 |  | 0.08 | 3.0E-02 |
| cg09517873 | 1 | 12656315 | *DHRS3* | Body | brown | 0.14 | -0.04 | 4.9E-04 |  | 0.17 | 3.0E-02 |
| cg11982546 | 8 | 56832362 | *LYN* | Promoter | blue | 0.74 | -0.05 | 2.5E-04 |  | 0.13 | 3.3E-02 |
| cg24154161 | 6 | 32820421 | *TAP1* | Body | pink | 0.15 | -0.06 | 3.3E-04 |  | -0.10 | 3.6E-02 |
| cg19111999 | 10 | 17270087 | *VIM* | Promoter | blue | 0.69 | -0.10 | 4.5E-04 |  | 0.08 | 4.4E-02 |

^a^ Location of CpGs in promoter regions, gene bodies, or 3' untranslated regions (3' UTRs).

^b^ Module membership by co-methylation analysis.

^c^ Average CpG methylation levels.

**Table S5.** Biological processes enriched by genes involved in 106 CpG methylation-gene expression pairs.

| GO Term | Count | Fold Enrichment | *P* value | *P*_Benjamini_ | Fold Enrichment |
| --- | --- | --- | --- | --- | --- |
| cell morphogenesis involved in differentiation | 8 | 6.52 | 1.97E-04 | 0.21 | *PRKCA, ATP2B2, LAMA5, BCL2, RXRA, PAX6, SOX9, DCLK1* |
| neuron development | 8 | 4.69 | 1.40E-03 | 0.56 | *PRKCA, ATP2B2, OPCML, BCL2, RXRA, PAX6, DCLK1, NTM* |
| cell morphogenesis | 8 | 4.47 | 1.85E-03 | 0.52 | *PRKCA, ATP2B2, LAMA5, BCL2, RXRA, PAX6, SOX9, DCLK1* |
| positive regulation of developmental process | 7 | 5.01 | 2.49E-03 | 0.52 | *PRKCA, TNFRSF1A, LYN, BCL2, PAX6, FOXO3, DDAH1* |
| cell recognition | 4 | 14.47 | 2.53E-03 | 0.45 | *OPCML, LAMA5, SCARB1, NTM* |
| regulation of locomotion | 6 | 6.22 | 2.59E-03 | 0.40 | *PRKCA, SERPINE2, LAMA5, BCL2, PAX6, SCARB1* |
| regulation of cell motion | 6 | 6.18 | 2.65E-03 | 0.36 | *SERPINE2, LYN, LAMA5, BCL2, PAX6, SCARB1* |
| cellular component morphogenesis | 8 | 4.01 | 3.40E-03 | 0.40 | *PRKCA, ATP2B2, LAMA5, BCL2, RXRA, PAX6, SOX9, DCLK1* |
| chemical homeostasis | 9 | 3.50 | 3.65E-03 | 0.38 | *PRKCA, MAFG, FXYD1, ATP2B2, GRIN2C, BCL2, GRIK3, SCARB1, FOXO3* |
| homeostatic process | 11 | 2.91 | 3.67E-03 | 0.35 | *PRKCA, MAFG, FXYD1, ATP2B2, NXN, LYN, GRIN2C, BCL2, GRIK3, SCARB1, FOXO3* |

**Table S6.** Clinical characteristics of 46 postmortem prefrontal cortex (PFC) tissue samples.

|  | AUD Cases  (n = 23) | Healthy controls  (n = 23) | *P-*value  (*t*-test) |
| --- | --- | --- | --- |
| Sex (Male), n (%) | 16 (69.6%) | 16 (69.6%) |  |
| Age (years) (mean ± S.D.) | 56 ± 9 | 57 ± 9 | 0.706 |
| Alcohol daily use (gram) (mean ± S.D.) | 165 ± 81 | 11 ± 9 | 5.8×10^-9^ |
| Postmortem interval (hours) (mean ± S.D.) | 39.7 ± 15.0 | 32.7 ± 13.4 | 0.103 |
| Brain weight (gram) (mean ± S.D.) | 1,380 ± 139 | 1,412 ± 135 | 0.434 |
| Brain pH (mean ± S.D.) | 6.58 ± 0.20 | 6.64 ± 0.27 | 0.371 |

**Table S7.** Primers designed for measuring methylation levels of six CpGs by bisulfite Sanger sequencing.

| CpGs (Genes) | Primers | Sequences (5'-3') | PCR |
| --- | --- | --- | --- |
|  |  |  | product size (bp) |
| cg01083716 | 1st-forward | TTATTAAGATTTTTTGGAAGAG | 436 |
| (*AGT*) | 1st-reverse | CCATCTTATCCTATTACTAAAAA |  |
|  | 2nd-forward | TTATTAAGATTTTTTGGAAGAG | 286 |
|  | 2nd-reverse | CTAACCCACAACTCAATTAC |  |
| cg23141914 | 1st-forward | AAAAATTATAGGTTTTTTTGTG | 226 |
| (*ALDH1L1*) | 1st-reverse | TTATTCTTTAAACTCCAACCTA |  |
|  | 2nd-forward | TGATTATATAATGGAGGAATTT | 206 |
|  | 2nd-reverse | TTATTCTTTAAACTCCAACCTA |  |
| cg02469186 | 1st-forward | TTTTTATTTGTTTGAGATGAAA | 443 |
| (*GABRA1*) | 1st-reverse | AAAATTTCAATTTCCACAATA |  |
|  | 2nd-forward | TGATTATAAAGTGTGTTTTTTAG | 283 |
|  | 2nd-reverse | TTTCCACAATAAACTATACATT |  |
| cg16086007 | 1st-forward | TAGTAGTGGGGTAAGTTAGAA | 419 |
| (*GRIN2C*) | 1st-reverse | TAAACTAATATTTAACAAACACAA |  |
|  | 2nd-forward | TAGTAGTGGGGTAAGTTAGAA | 349 |
|  | 2nd-reverse | AAACACAACAACATCTAAAA |  |
| cg09656389 | 1st-forward | TTAGTAGGTTTGGAGAGATTTT | 442 |
| (*PAX6*) | 1st-reverse | CTCCAACCCCTACTTTAAC |  |
|  | 2nd-forward | AGAGAGGTAGTTGGTTAGTTTAAG | 182 |
|  | 2nd-reverse | TACTTTAACCTTCCTTAACCC |  |
| cg16610086 | 1st-forward | TAATTGTTGAAATAGAGGTATG | 422 |
| (*SLC1A3*) | 1st-reverse | TAATTAATCTTAACACACCAAA |  |
|  | 2nd-forward | TAATTGTTGAAATAGAGGTATG | 382 |
|  | 2nd-reverse | ATTTTACTCCTTAAACCACTT |  |

The Sanger sequencing method was used to validate methylation levels (obtained from methylation array-based assays) of six CpGs that showed significant methylation differences between 23 AUD cases and 23 healthy controls by paired *t*-tests. About 500 ng of genomic DNA was treated with bisulfite reagents included in the EZ DNA Methylation-Gold Kit (Zymo Research, Orange, CA, USA) according to the manufacturer’s protocol. Nested PCR was performed to obtain amplicons for direct DNA sequencing. For the 1^st^ PCR, 20 µL of PCR reactions were prepared, including 10× PCR buffer, 1.5 mM Mg^2+^, 10 ng of bisulfite-treated genomic DNAs, and 0.5 µM of the 1^st^ pairs of primers. PCR conditions were 94°C 3 min (denaturation), 35 cycles of 94°C 30 sec, 50°C 30 sec, and 72°C 30 sec, then 72 °C 10 min (extension). For the 2^nd^ PCR, PCR reactions were similar as those for the 1st PCR, except 1 µL of 100-fold diluted 1^st^ PCR products (as templates) and the 2nd pairs of primers were used. Thermal conditions were 94°C 3 min (denaturation), 35 cycles of 94°C 30 sec, 52°C 30 sec, and 72°C 30 sec, then 72 °C 10 min (extension). Detailed information regarding DNA sequencing and data analysis were described in our previous study^10^. Average methylation levels were calculated for the 46 samples. PCRs were failed in the amplification of DNA sequences containing CpG cg16086007 in *GRIN2C* and cg01083716 in *AGT*.

**Table S8.** Top 10 pathways enriched by 104 genes mapped by the top 154 differentially methylated CpGs identified in female AUD subjects

| GO ID | GO Term | Count | Fold Enrichment | P Value | Benjamin | Genes |
| --- | --- | --- | --- | --- | --- | --- |
| GO:0006997 | nucleus organization | 3 | 12.6 | 2.3E-02 | 1.0E+00 | DFFB, CECR2, TSSK6 |
| GO:0022411 | cellular component disassembly | 3 | 12.4 | 2.4E-02 | 1.0E+00 | DFFB, CECR2, XRN2 |
| GO:0006355 | regulation of transcription, DNA-dependent | 15 | 1.8 | 2.4E-02 | 1.0E+00 | NDN, INS-IGF2, TEAD1, NOSTRIN, ZNF331, LASS4, ZNF225, FOXF1, ZNF222, GATAD2A, JAK3, TLX3, ALX4, MLL4, PEG3 |
| GO:0051252 | regulation of RNA metabolic process | 15 | 1.8 | 2.9E-02 | 1.0E+00 | NDN, INS-IGF2, TEAD1, NOSTRIN, ZNF331, LASS4, ZNF225, FOXF1, ZNF222, GATAD2A, JAK3, TLX3, ALX4, MLL4, PEG3 |
| GO:0006308 | DNA catabolic process | 3 | 10.9 | 3.0E-02 | 9.9E-01 | DFFB, CECR2, XRN2 |
| GO:0009743 | response to carbohydrate stimulus | 3 | 10.9 | 3.0E-02 | 9.9E-01 | PFKL, COLEC12, SELS |
| GO:0040029 | regulation of gene expression, epigenetic | 3 | 8.5 | 4.7E-02 | 1.0E+00 | INS-IGF2, GATAD2A, MLL4 |
| GO:0050995 | negative regulation of lipid catabolic process | 2 | 33.6 | 5.7E-02 | 1.0E+00 | INS-IGF2, PDE3B |
| GO:0045935 | positive regulation of nucleobase, nucleoside, nucleotide and nucleic acid metabolic process | 7 | 2.4 | 6.1E-02 | 1.0E+00 | INS-IGF2, FOXF1, ZXDC, TEAD1, JAK3, ALX4, MLL4 |
| GO:0045449 | regulation of transcription | 18 | 1.5 | 6.5E-02 | 1.0E+00 | NDN, INS-IGF2, ZXDC, CENPF, TEAD1, NOSTRIN, ZNF331, LASS4, ZNF225, FOXF1, ZNF222, GATAD2A, JAK3, TLX3, ALX4, MLL4, XRN2, PEG3 |


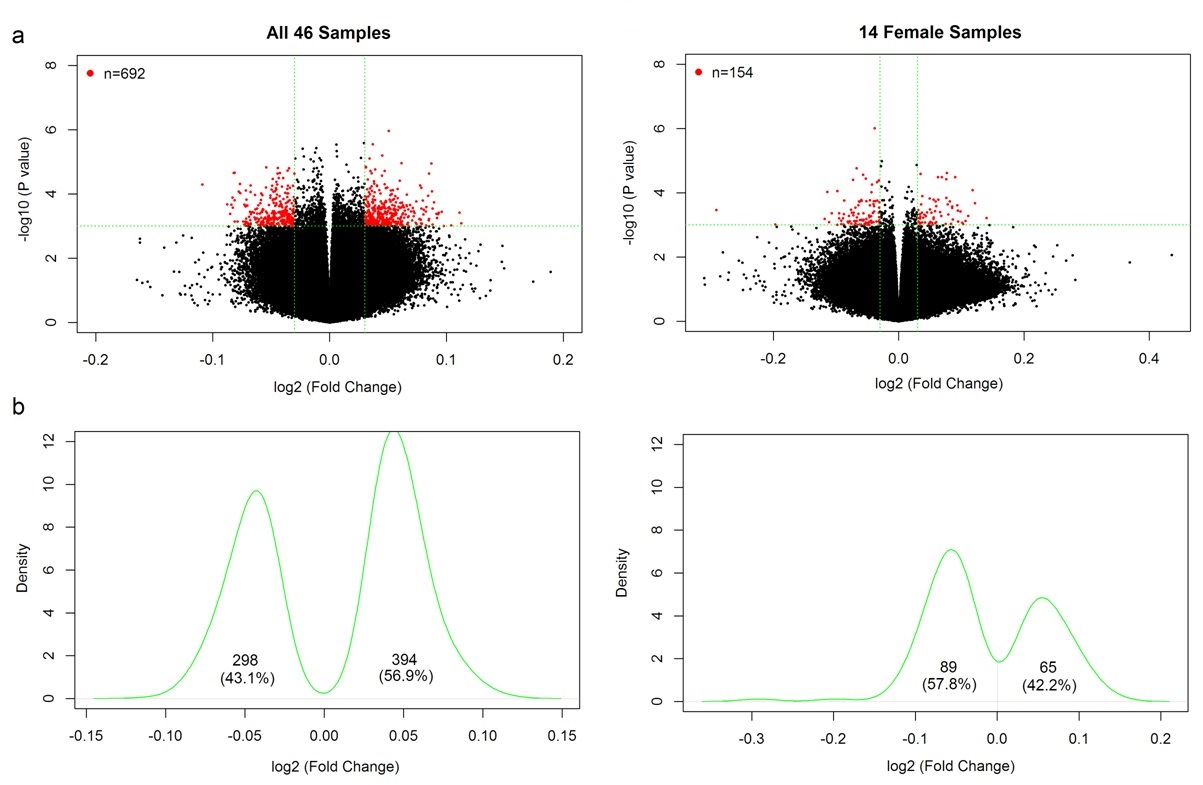


**Figure S1.** Differentially methylated CpGs in all or female subjects with alcohol use disorders (AUDs).

(a) Volcano plot of the effect size [log_2_ (fold changes)] against -log_10_ (*P* values) of 434, 015 CpGs in all 23 pairs of samples (left) or seven pairs of female samples (right). The red dots represent CpGs with *P* ≤ 1.0 × 10^-3^ (the horizontal green dash line) and the absolute value of log_2_ (fold change) > 0.03 (two vertical green dash lines), and the black dots represent CpGs with *P* > 1.0×10^-3^ and the absolute value of log_2_ (fold change) ≤ 0.03.

(b) Kernel density plot of log_2_ (fold changes) of CpGs (represented by red dots) in all 23 pairs of samples (left) or seven pairs of female samples (right).


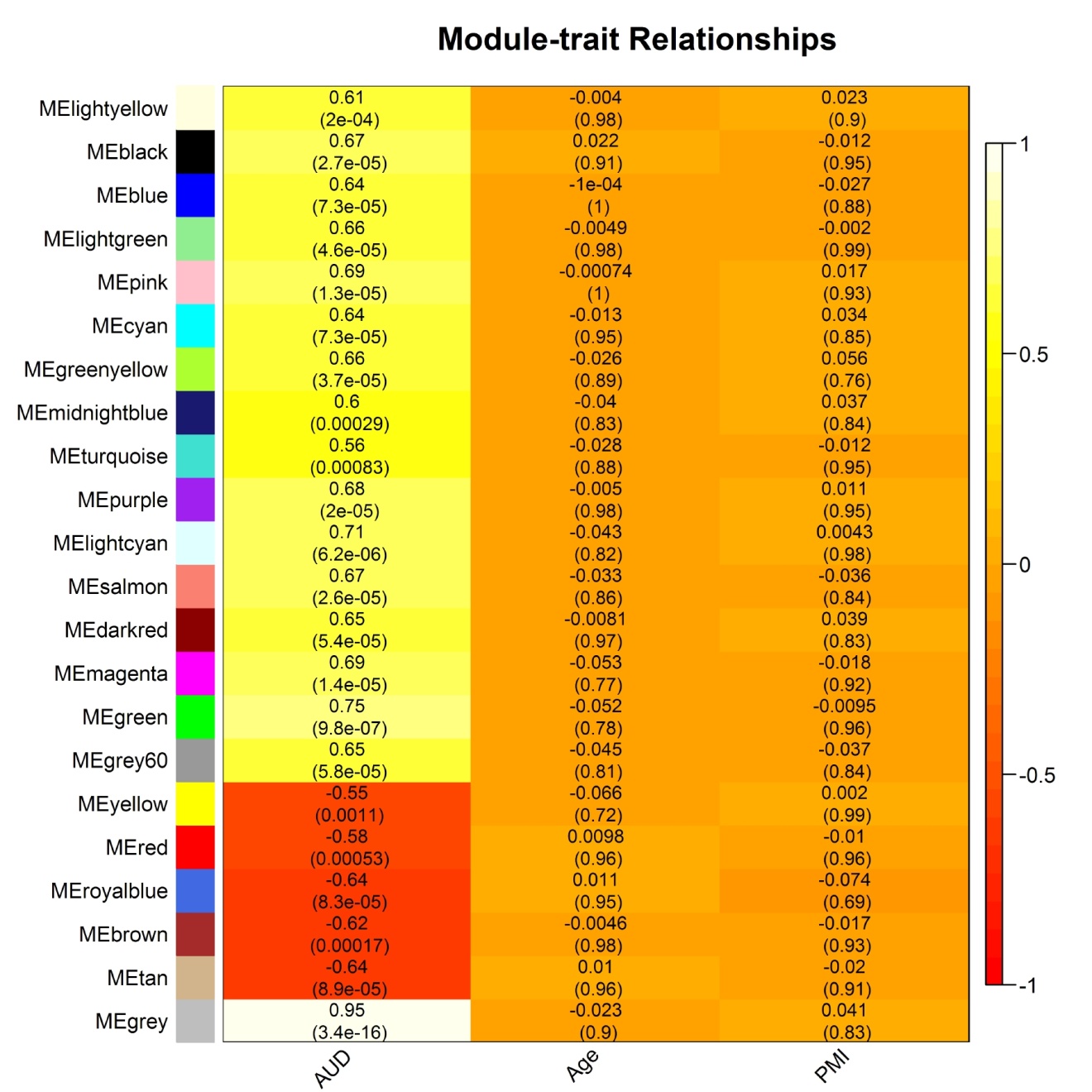


**Figure S2.** Co-methylation analysis of nominally significant CpGs identified in male subjects with alcohol use disorders (AUDs).


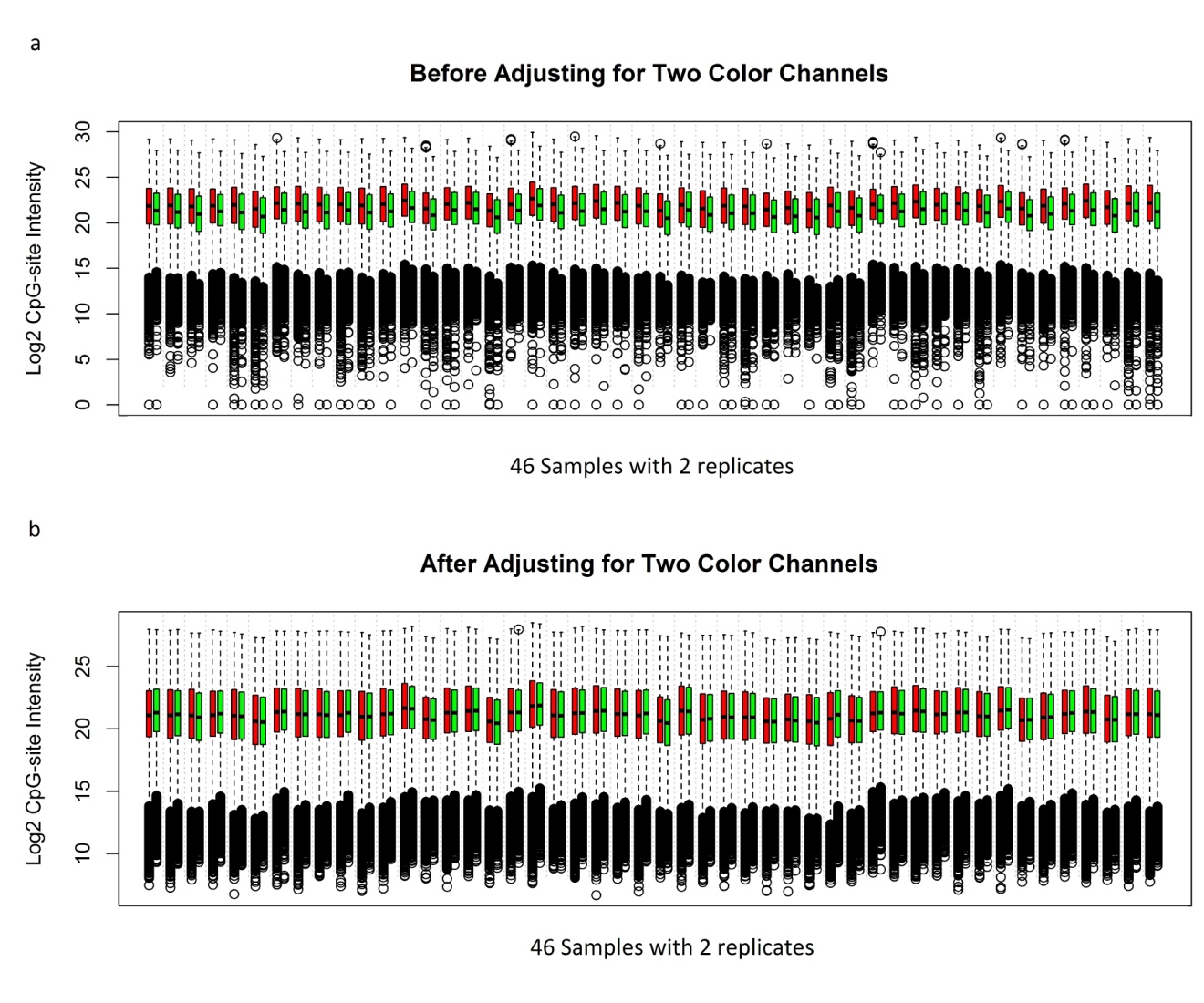


**Figure S3.** Distribution of CpG levels determined by two color channels (a) before and (b) after adjusted by the R package *lumi*.


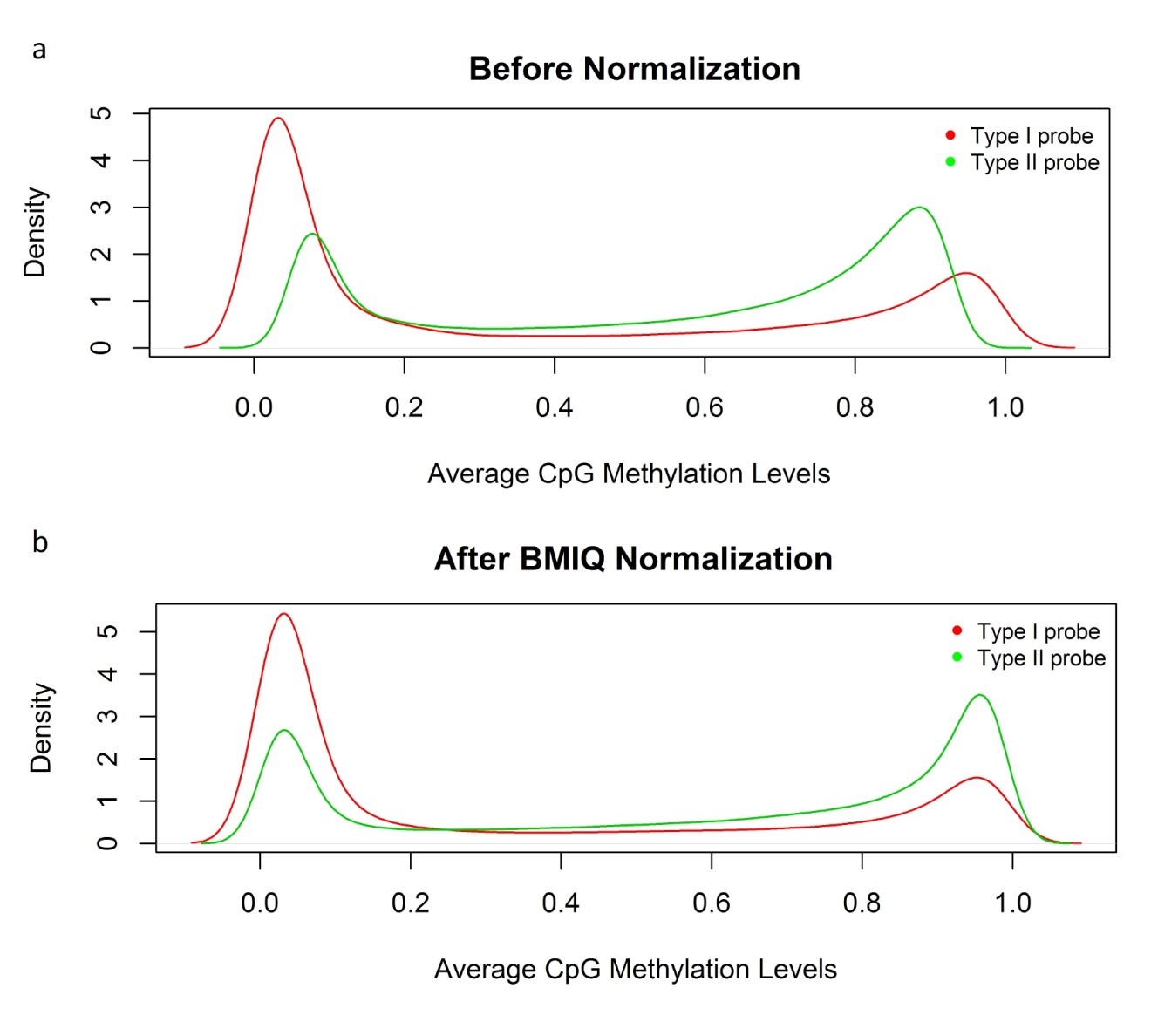


**Figure S4.**  Distribution of CpG methylation levels determined by two types of probes across all 46 samples.


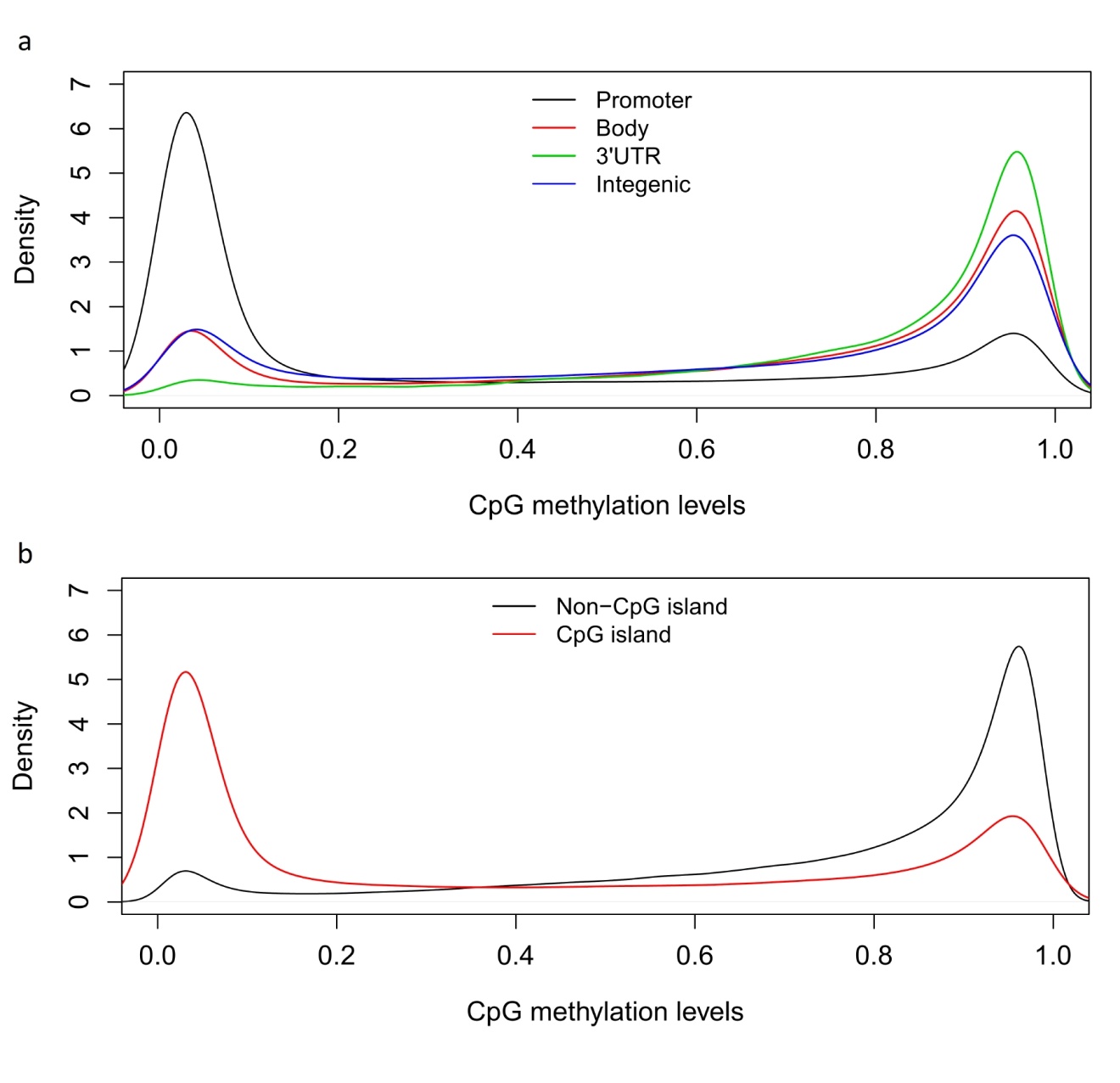


**Figure S5.** Distributions of methylation levels of 434,015 CpGs across the genome.


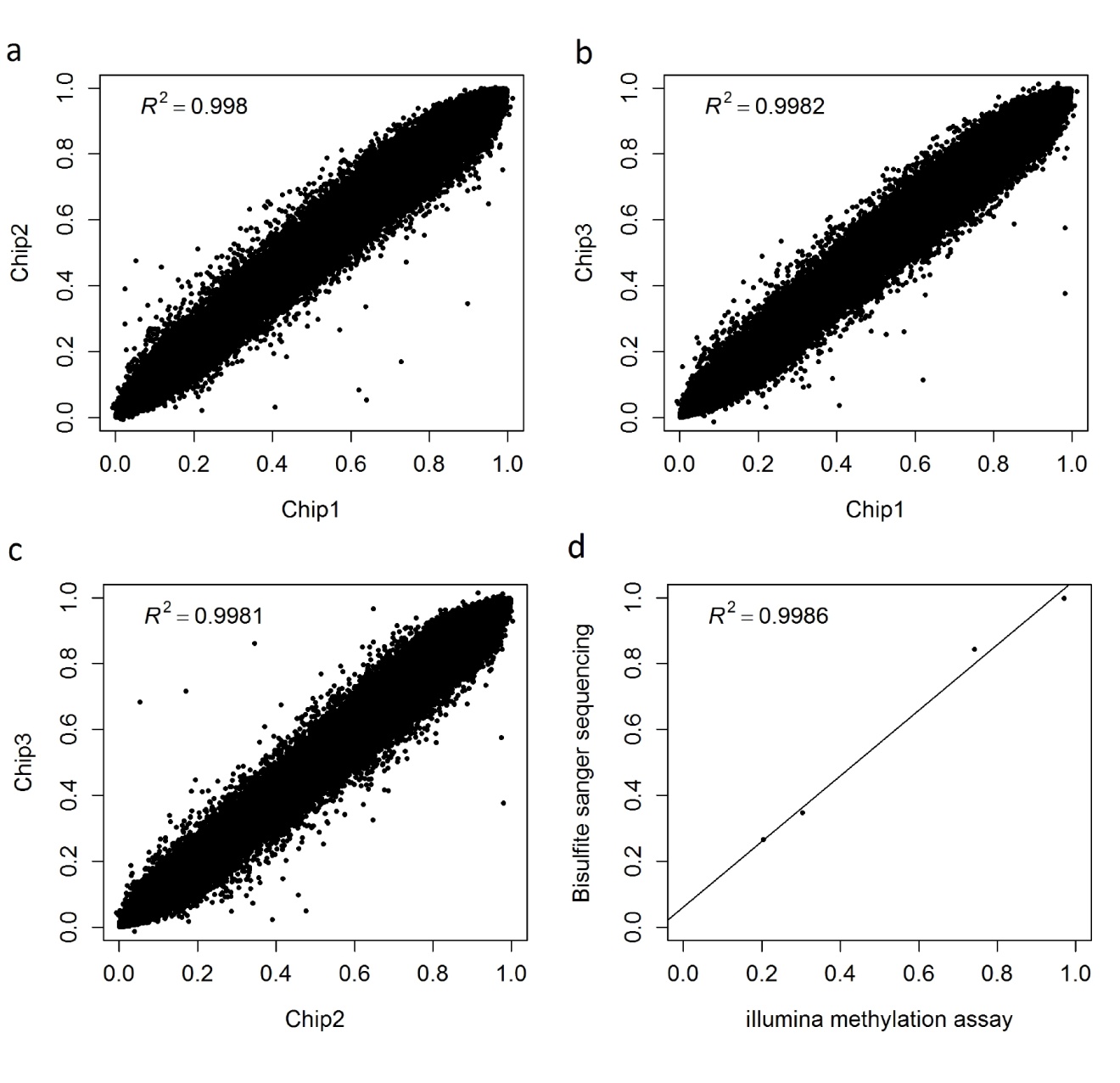


**Figure S6.** Assessing reproducibility of Illumina Infinium HumanMethylation450 BeadChip assays and validating methylation levels of six CpGs using bisulfite Sanger sequencing.
